# Enumeration Class of Polyominoes Inscribed in James Abacus and Related ECO

**DOI:** 10.12688/f1000research.172910.1

**Published:** 2026-01-06

**Authors:** Eman F. Mohommed, jalal Abd Jassim

**Affiliations:** 1 mathematic, Mustansiriyah University Department of Mathematics, Baghdad, Baghdad Governorate, Iraq

**Keywords:** Polyominoes, Class, Enumeration, Succession-rule, Abacus diagram

## Abstract

This paper studies a new family of polyominoes. The family is defined through a graphic representation called James diagram, also known as the nested chain abacus. This form is referred to as the Ω-nested Abacus. The proposed class is defined by geometric constraints satisfied by sets of rows, columns, and chains. Through the application of combinatorial approaches and variable generating function, we enumeration of this new class of polyominoes. During the new construction process transformation is formulates which plays a crucial role in the enumerate of a family of classes. To further extend this study generating function for theses class are formulated using an enumeration method known as the Enumeration Combinatorial Object (ECO) technique. ECO method, derived from a smaller one though a series of local expansion. Theses expansion are systematically defined by succession rule which can subsequently be expressed as a functional equation for the generating function.

## Introduction

**Figure 1.**
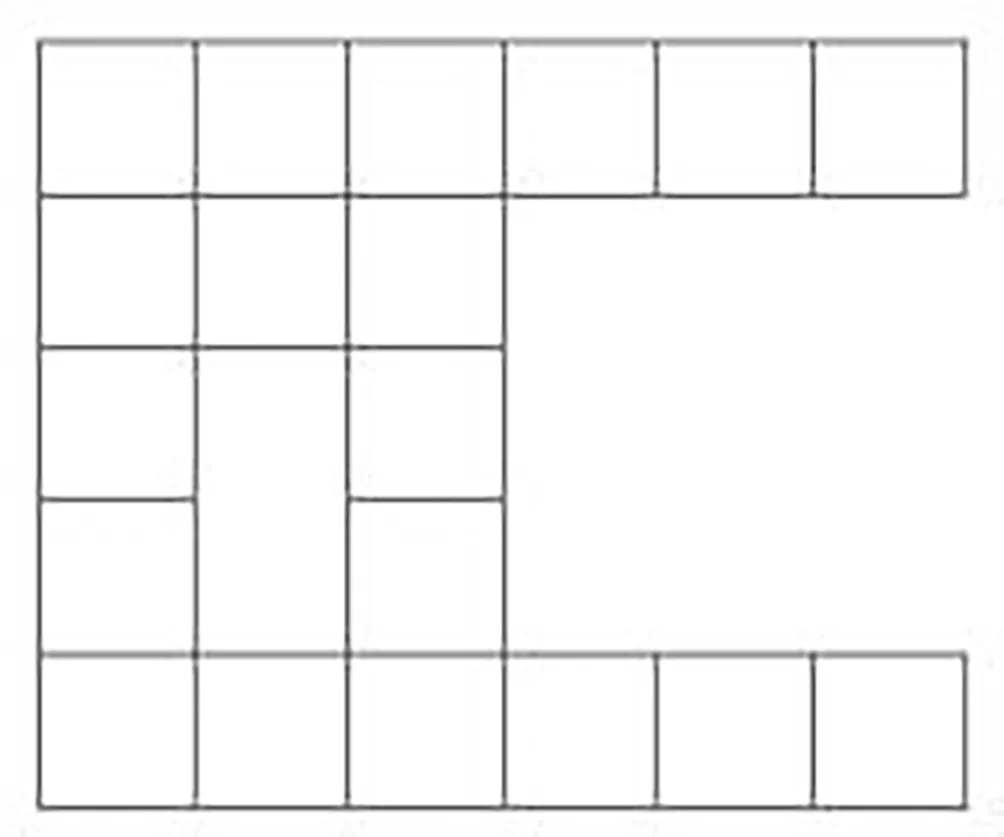
22-polyominoes (polyominoes with 22 connected ominoes).

Polyominoes are finite configurations composed of unit squares, known as ominoes, that are joined edge to edge to form a connected interior, as illustrated in Figure 1.

**Figure 2.**
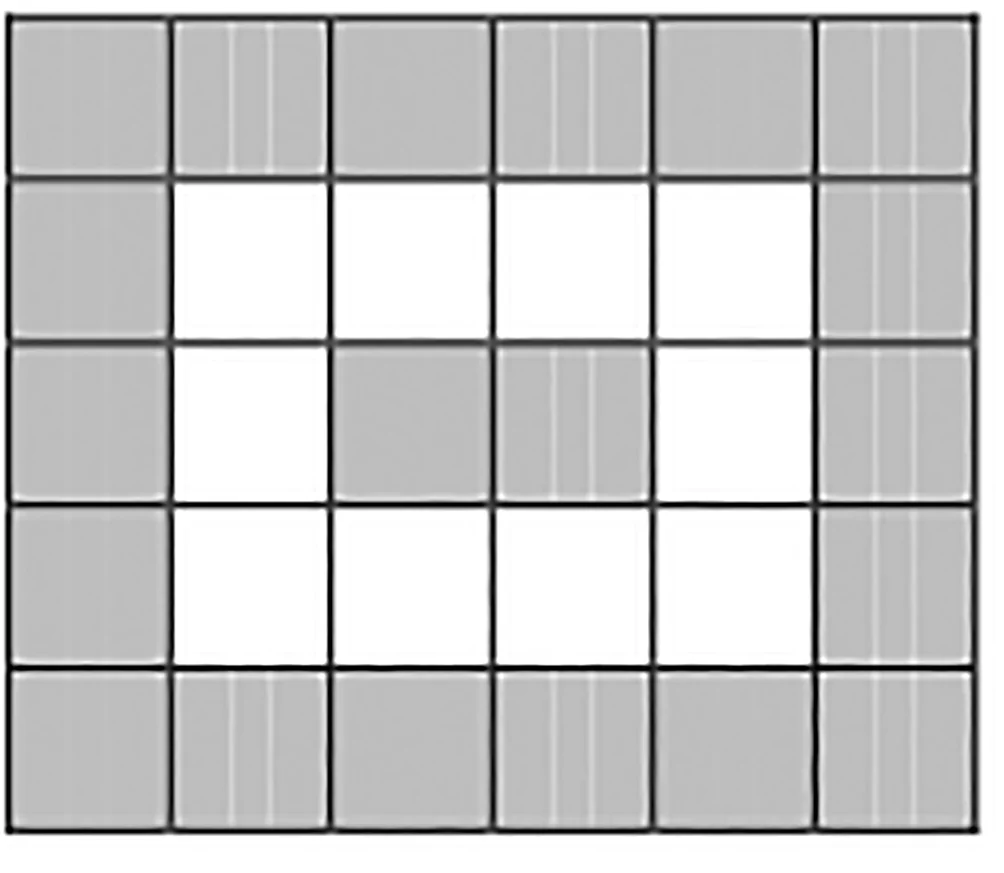
Nested chain abacus with 6 columns, 5 rows and 3 chains.

An n -polyomino (a polyomino consisting of n ominoes) is defined up to translation, and the concept is commonly attributed to Golomb. ^1^ In contemporary research, n-polyominoes have attracted significant attention from computer scientists, physicists, mathematicians, and biologists. Despite extensive studies, enumerating n-polyominoes remains a challenging and unresolved problem in combinatorial geometry, representing one of its most fundamental open questions. ^2– 5^ There is no closed-form equation for *n*-polyominoes, and the problem has been solved up to *n* < 56. ^3^ No closed-form expression for the enumeration of n -polyominoes is currently known. Due to the complexity of this problem, several simpler subclasses of n -polyominoes have been formulated and extensively examined in the literature. ^6– 8^ E.F. provide a new representation of *n*-polyominoes (Plyominoes with *n* ominoe) using one of the graphical representations of partition (James-diagram) called nested chain abacus (N.C.A.) with b -columns and d -rows. ^9^ The Nested Chain Abacus (N.C.A.) offers a novel framework for representing any n connected omino, including empty ones (holes), through the use of a beta number. In this new representation, n -polyominoes are organised into a series of nested chains. ^10^ A Nested Chain Abacus (N.C.A.) is an n -polyomino inscribed within a James diagram, consisting of both outer and inner chains. The inner chains are numbered from 1 to n , where n is a positive integer, and chain 1 is the innermost chain. No intersections occur among any of the chains. Through this new representation, and for the first time, each n-polyomino has been systematically associated with a unique code. Next Figure 2 illustrates an example of an N.C.A. with six columns, five rows, and three chains.

## Terminologies and definition

This section introduced some importuned definitions:

Definition 1. A partition of any integer, k , is a sequence of integers $\lambda_{1,}\lambda_{2,}\ldots ,\lambda_{n}$ such that $\lambda_{1}\;\geq \;\lambda_{2}\;\geq \;\ldots \;\geq \;\lambda_{n}$ and $\sum_{i=1}^{n}\lambda_{i}=k$ .

Example 1.
λ=(6,6,3,2,1,1) is a partition of 19

Definition 2. A sequence of positive numbers { $\;\beta_{1},\beta_{2},\ldots ,\beta_{k}\}\;$ called beta number such that $\;\beta_{i}=\lambda_{i}+k-i\;$ and 1≤i≤k . λ

Example 2. Consider of Example 1, λ=(4,4,2,2,1,1,1,1) then beta number of λ is

$$\;\beta_{i}=\lambda_{i}+b-i,\;\text{where}\;i=1,2,3,4,5,6$$

$$\;\beta_{1}=\lambda_{1}+b-1=4+8-1=11$$

$$\;\beta_{2}=\lambda_{2}+b-2=4+8-2=10$$

$$\;\beta_{3}=\lambda_{3}+b-3=2+8-3=7$$

$$\;\beta_{4}=\lambda_{4}+b-4=2+8-4=6$$

$$\;\beta_{5}=\lambda_{5}+b-5=1+8-5=24$$

$$\;\beta_{6}=\lambda_{6}+b-6=1+8-6=3$$

$$\;\beta_{7}=\lambda_{7}+b-7=1+8-7=2$$

$$\;\beta_{8}=\lambda_{8}+b-8=1+8-8=1$$

Thus beta number sequence is {1,2,3,4,6,7,10,11} .

Definition 3. James Abacus is a graphical representation of a partition of any integer number using beta numbers.

**Figure 3.**
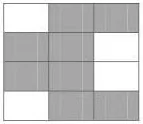
James’s Abacus with 3 columns, 4 rows and 6 beta.

Consider Example 1, the James Abacus with 1,2,3,4,6,7,10,11 beta position as show in next Figure 3.

**Figure 4.**
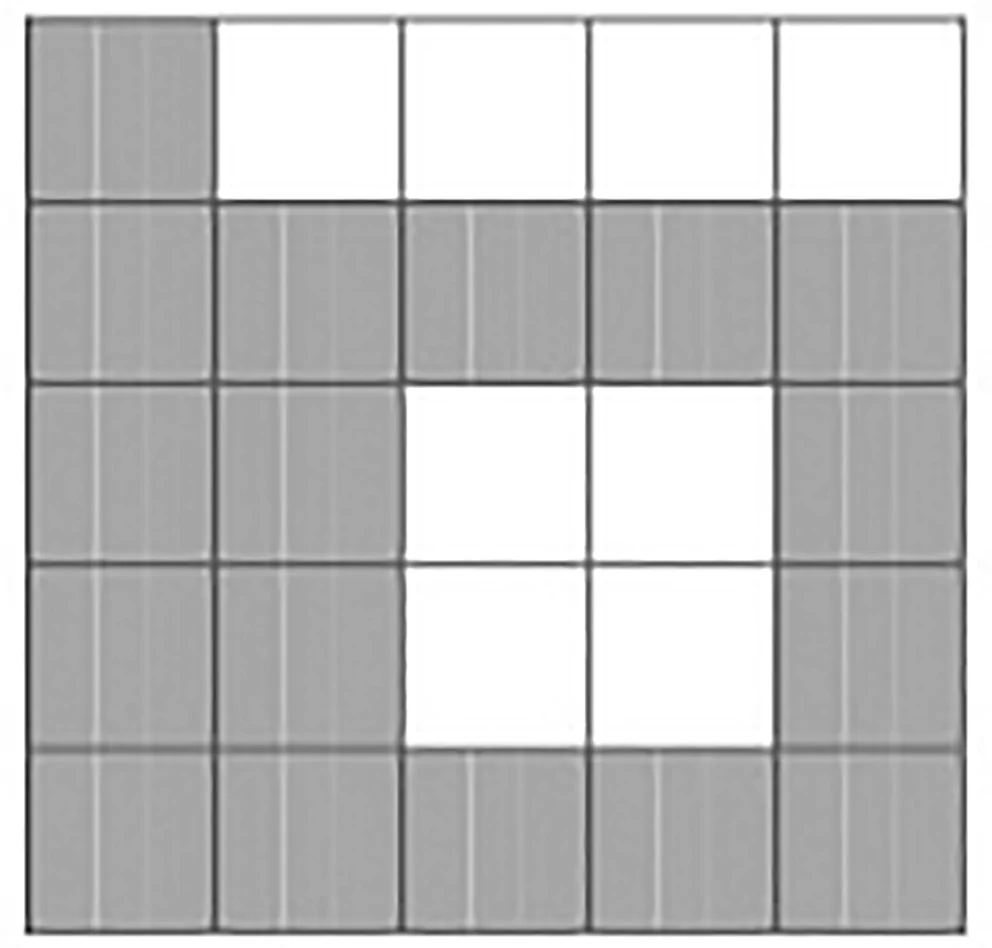
N.C.A. with 5 columns ( b=5 ) and 3 chains represented of 94, where the beta number sequence is { 0,5,6,7,8,9,10,11,14,15,16,19,20,21,24}.

Next Figure 4 gives an example of N.C.A. with 3 chains represented of 94, where the beta number sequence is { 0,5,6,7,8,9,10,11,14,15,16,19,20,21,24}.

Definition 4: A connected chain is a chain with only beta positions.

**Figure 5.**
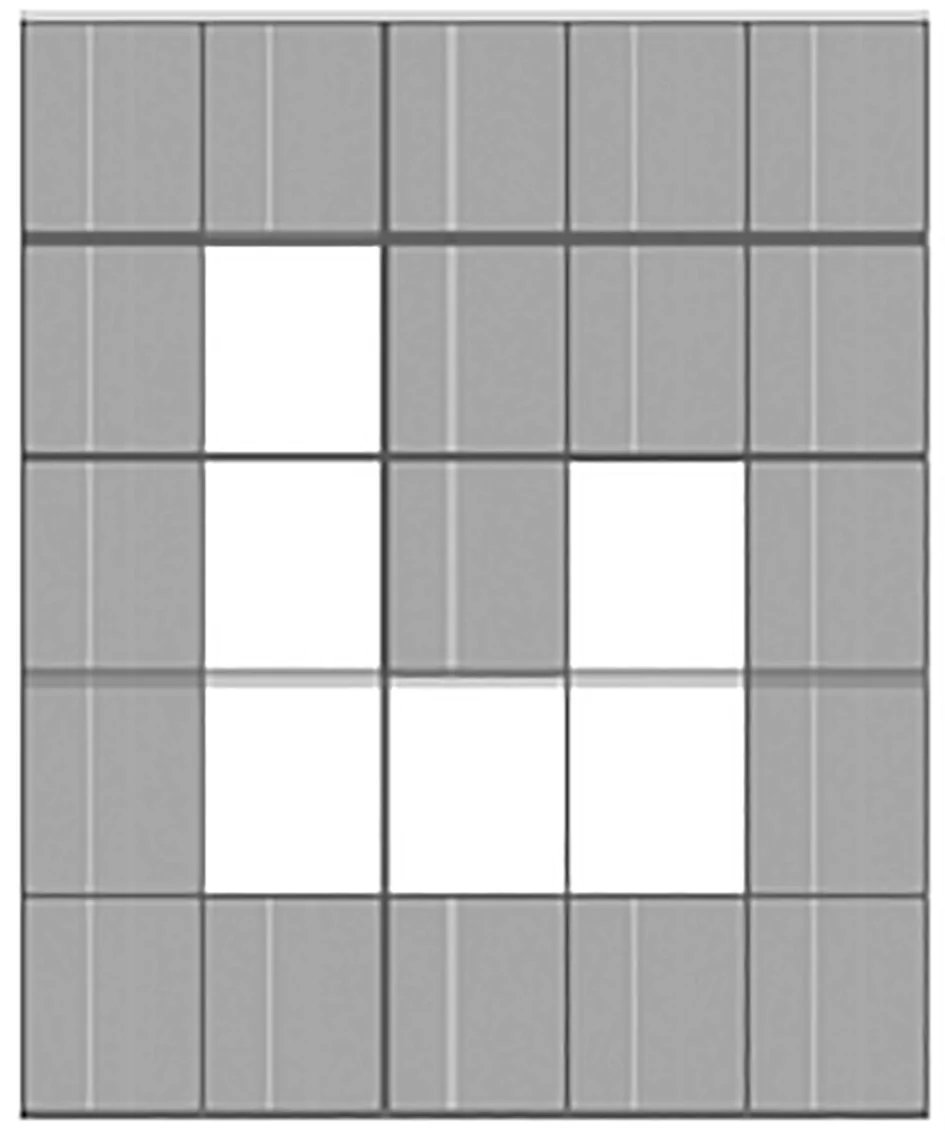
N.C.A. with 1 connected chains (chain 3).

Next Figure 5 gives an example of N.C.A. represented by a beta sequence {0,1,2,3,4,5,7,8,9,10,12,14,15,19,20,21,22,23,24} with one connected chain (chain 1).

Definition 5. Let $\;\beta_{i},\;\beta_{j}$ be two beta numbers in N.C.A then $\;\beta_{i},\;\beta_{j}$ are connected iff ^4^

1-
$\left|\;\beta_{i}-\beta_{j}\;\right|=1$ if $\;\beta_{i},\;\beta_{j}$ Located in the same row.
2-
$\left|\;\beta_{i}-\beta_{j}\;\right|=b\;\text{if}\;\;\beta_{i},\;\beta_{j}\;\text{Located in the same column}$ .

Consider Figure 4; the set of beta {0,5,6,7,8,9,10,11,14,15,16,19,20,21,24} is a connected beta set.

Definition 6. Each nested chain abacus (N.C.A) with b columns is called a polyamines if every two beta numbers are connected.

Definition 7.
Ω - nested Abacus is a N.C.A with b columns and d rows satisfy the following condition.

1- chain 1 with only one position
  a) If chain 1 has a beta position, then chain 2 has at least 5 beta positions.
  b) If chain 1 with empty beta position, then chain 2 has k beta positions, where 1≤k≤8

2- Chain number o are connected chain where o is an odd number.
3- Chain number e contains beta position as well as empty beta where o is an odd number.

**Figure 6.**
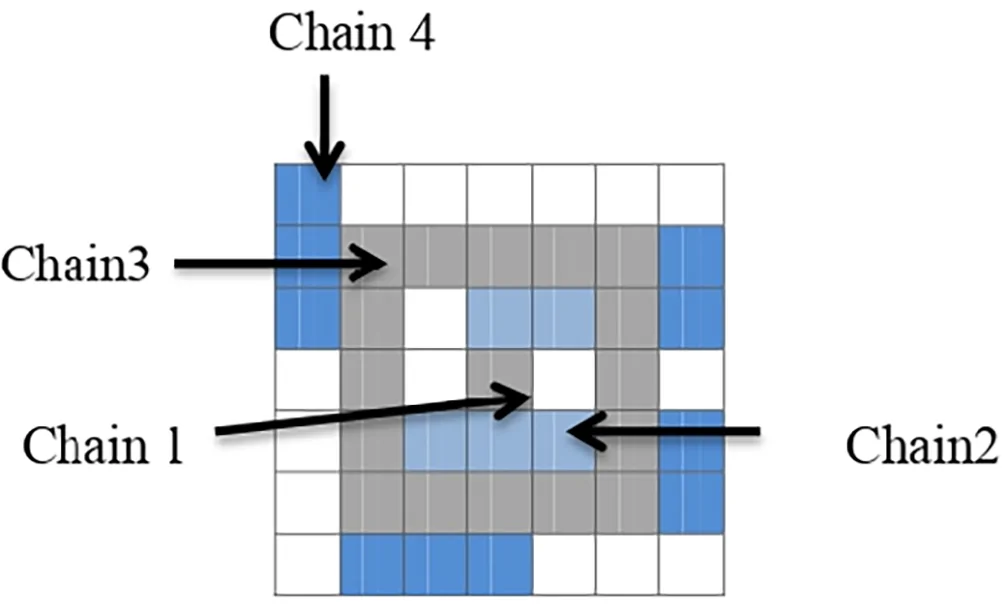
Ω - nested Abacus with four chains.

Figure 6 gives an example of Ω - nested Abacus with four chains, where

Not Every polyamines inscribed in a nested chain-abacus is Ω - nested Abacus.

Figure 4 given an example of N.C.A. but not Ω - nested Abacus, while Figure 6 given an example of Ω - nested Abacus.

Definition 8. Single – beta transformation (SPT) is a beta movement in chain i is

$$⟶\begin{array}{c} \overset{`}{\beta }\;=\left\{\left(m-1\right)b+\left(\;j-1\right)\right\}\\ \left\{ \begin{array}{cc} \overset{`}{\beta }\;=\left(m-1\right)b+\left(j-2\right) & \text{if}\;i+1\;\leq \;m\;\leq \;\left(r-i+1\right),\;j=e-i+1\\ \overset{`}{\beta }\;=\mathrm{mb}+\left(j-1\right) & \text{if}\;i\;\leq \;m\;\leq \;\left(r-i\right),\;j=i\\ \overset{`}{\beta }\;=\left(m-1\right)b+j\; & \text{if}\;m=i,\;i+1<j\;\leq \;e-i+1\\ \overset{`}{\beta }\;=\left(m-2\right)b+\left(j-1\right) & \text{if}\;m=r-i+1,\;i\;\leq \;j<e-i. \end{array}\right. \end{array}$$

With d rows, b columns where β={(m−1)b+(j−1)} is the beta number in column number j and row number m .

Remark 10. The maximal number of transformations in chain n is equal to the number of positions in the chain, where n>1 .

**Figure 7.**
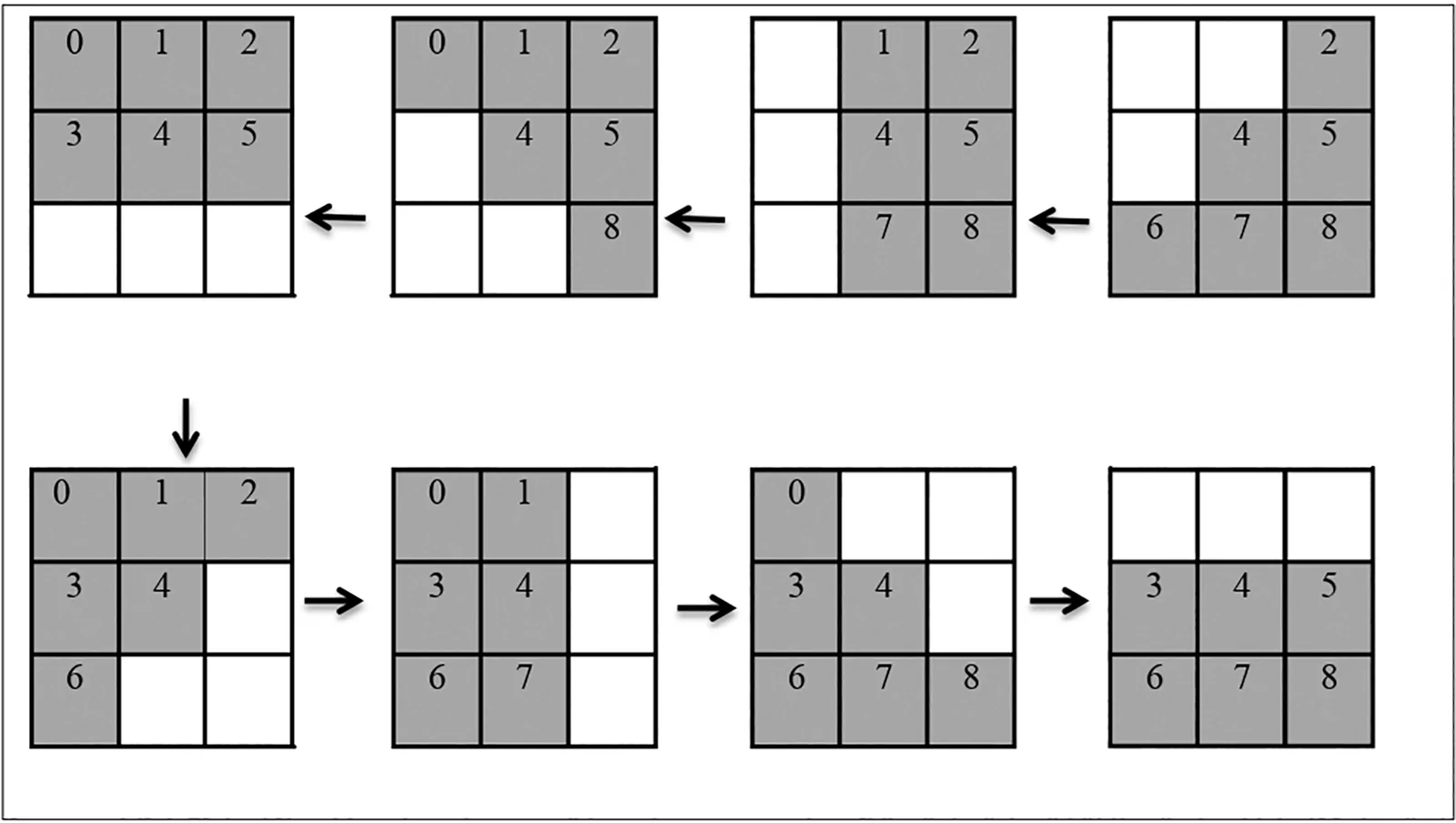
Illustrates Single – beta transformation (SPT) on Ω - nested Abacus.

Figure 7 illustrates Single – beta transformation (SPT)

Not.

1- SSpt is a SPT transformation in one chain.
2- MSpt is a SPT transformation in all chains.

## Enumeration of the Ω - nested Abacus class

Next, the enumeration of Ω-nested Abacus with b columns and d rows is presented.

Lemma 10. Let of Ω-nested Abacus be Abacus with b rows and b columns, then the number of positions in chain n is ( $\left(n-1\right)\;2^{3})$ where n>1 and b is an odd number.

Proof. Based on, ^8^ chain 1 consists of one position. To build the second chain, we add a position above, below, to the right, and to the left of chain number 1(consisting of one position), as well as the far right and far left from the top and the far right and left from the bottom of chain number 1. Then the number of positions in chain 2 is equal to $2^{3}$ . Based on theorem 2.5.11, ^13^ the difference between chain i and chain $\left(i+1\right)=2^{3}$ . Thus, the number of positions in chain n is ( $\left(n-1\right)\;2^{3})$ where n>1 .

Lemma 11. The number of chains in the Ω-nested Abacus is $\frac{b+1}{2}$ where b is the number of columns in Ω-nested Abacus.

Proof. See Theorem 2.5.21. ^13^

Theorem 12: Let a be the number of even chains, then the number of Ω-nested Abacus generating by employ MSPT- Transformation is:

∑(b+14(n−1)23)

Proof. Based on Lemma 10, the number of positions in chain *n* is $\left(n-1\right)2^{3}$ by Lemma 11 and Definition 8, there are $\frac{\;p+1}{4\;}$ chain with beta and empty beta position in Ω-nested Abacus. The maximal number of transformations in chain n is equal to $\left((n-1\right)\;2^{3})$ . Since each move generates a new Abacus (polyominoes), thus there are $\left((n-1\right)\;2^{3})$ of Ω-nested Abacus generated by employing MSpt transformation. As a result of this, there are (b+14(n−1)23) Ω--nested Abacus in chain n .

Corollary 13: The number Ω-nested Abacus with b columns and d rows in chain *i* is

2d+2b–4(2i–1)

Proof. Based on theorem 2.5.9 ^13^ Chain i have 2d+2b−4(2i–1) position then the each beta can be move

2d+2b–4(2i–1) so 2d+2b–4(2i–1) shapes can be construct after application MSPT.

Theorem 14: The number Ω-nested Abacus after the application MSPT transformation:

$$\pi \left(\frac{p+1}{4}\right)2d+2p\_4\;\left(2i-1\right)$$

proof. Based on (Corollary 1), then are 2r+2e–4(2i–1) position in chain i

since we have $\frac{p+1}{4}$ chain with empty beta, then the number Ω-nested Abacus is

$$\pi \left(\frac{p+1}{4}\right)2d+2p\left(2i-1\right)4.$$

## Generating function with respect to chains

In this section, the method described in ^11, 12^ is employed to enumerate the Ω-nested Abacus class, representing polyominoes inscribed within a James diagram. The ECO (Enumerating Combinatorial Objects) method has previously been applied to the enumeration of various polyomino classes. ^9, 10^ This approach is based on a *succession rule.*

## Succession rule

It relies on a well-defined notion of size, such that the number of objects of size n is finite for any positive integer n. There exists exactly one object of size 1. A generating tree for any class is an infinite rooted tree whose vertices are the objects of the class, each appearing exactly once in the tree, and such that objects of size n are at level n . If we give a class ϑ of discrete objects and a parameter ρ on ϑ , where $\vartheta_{n}=\left\{x\;\in \;\vartheta \;:p\left(x\right)=n\right\}$ . Let θ be an operator which constructs every object $Y\;\in \;\vartheta_{n}$ from another $Y\;\in \;\vartheta_{n}$ such that Y get from exactly one $X\;\in \;\vartheta_{n-1}$ . then, we have a recursive construction of all the elements of Ω ; from this, in turn, we will some of the time deduce a functional equation verified by the creating work of ϑ . The development can be represented through a growth tree, where vertices corresponding to objects that share the same measurement with respect to the parameter p are positioned at the same level. The children of a given vertex correspond to the objects derived from it, and each vertex is labeled according to the number of its offspring. This values the richness of the nodes (and of the comparing object). If the names of the children of a hub named ( *k*), as it were, depend on the *k*, able to speak to the developing stage of the tree by implies of the following (called succession-rule).

$$\vartheta =\left\{ \begin{array}{ccc} \left(X\right) & & \\ \left(Y\right) & \to & \left(y_{1}\right)\left(y_{2}\right)\ldots \left(y_{i}\right) \end{array}\right.$$

Where X is the tag of the root and $\left(y_{i}\right)$ is the tag of the k-th son of a node tagged i , a succession-rule can be represented by a generating tree.

The purpose of this section is to demonstrate how variations in succession rules can influence the structure of the corresponding generating function. Moreover, describe the class of Ω-nested Abacus polyominoes, which are enumerated by factorial numbers with respect to their coordinated height. Furthermore, examine the succession rule that represents the growth pattern of this class as derived using the ECO method. From the succession rule, it produces a sequence, ${\left\{g_{n}\right\}}_{n}$ , of positive integers, such that $g_{n}$ is the number of nodes in level *n* of the generating tree, and the generating function of the generating tree is denoted by $g_{\vartheta }=\sum g_{n}\;x^{n}$ (Bacchelliet al., 2010). In this work, our succession rule will be constructed starting from a single chain (chain with a beta), which will grow step by step by adding *C _i_* beads, where *C _i_* is the number of positions in chain *i,* as shown in Figure 4.10, which illustrates the number of nested chain abacuses in levels 1 and 2. A label is assigned containing all the essential information necessary to describe how the Ω-nested Abacus class evolves within the generating tree.

## Generating tree

• First level of the generating tree of ϑ begins with Ω - nested Abacus class consist of a single beta ( $\beta_{s})$ where $\left(\beta_{s}=\frac{b^{2}-1}{2}\right)$ .
• Second level $L_{2}$ we add one of the following beta number sets called $\beta_{L_{2}}$ where
$$\beta_{L_{2}}=\left\{\beta_{s}+1,\;\beta_{s}-1,\;\beta_{s}+b,\;\beta_{s}-b,\;\beta_{s}+b+1,\;\beta_{s}+b-1,\;\beta_{s}-b+1,\;\beta_{s}-b-1\right\}$$

Such that b is the number of columns, so the 8 of Ω **-** nested Abacus class called $\Omega_{L_{2}}$ .

**Figure 8.**
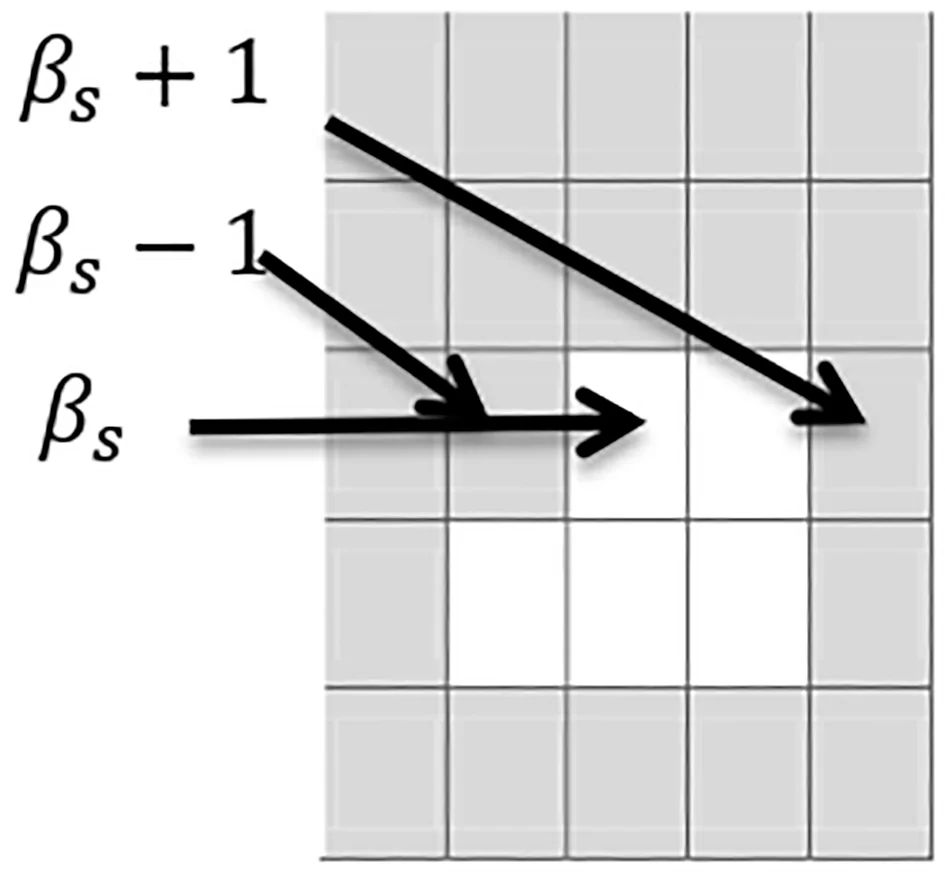
$\;\beta_{L_{2}}$ set in Ω **-** nested Abacus class.

**Figure 9.**
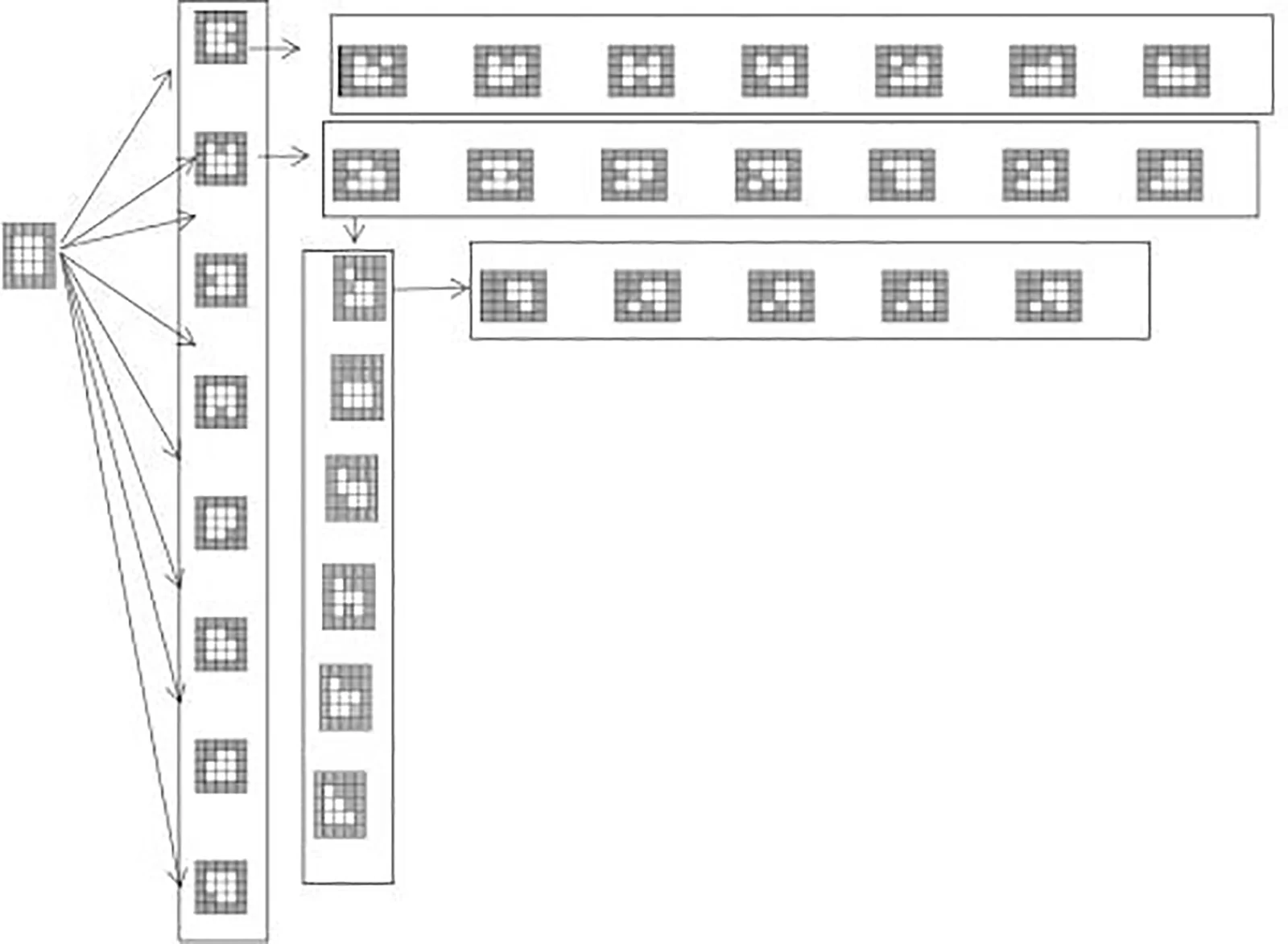
First and second level of ϑ using Ω - nested Abacus class with 5 columns, 5 rows and 3 chain.

• Third level $L_{3}$ in this level e take one of $\Omega_{L_{2}}$ **class** add one of the $\beta_{L_{2}}$ set, so the 7 of Ω **-**nested Abacus class called $\Omega_{L_{3}}$ and then, as shown in Figure 8 and Figure 9.

## The succession rule of Ω -nested Abacus (polyominoes)

Let Ω **-** nested Abacus be a polyominoes class with b columns and d rows, where i be a number of chains. The initial set began with two chains and a singleton beta ( $\beta_{s})$ . The Ω - nested Abacus class can be described, depending on the generating tree, by the overview rule:

$$\vartheta =\left\{ \begin{array}{ccc} \left(1\right) & & \\ \left(8i-8\right) & \to & \left(1\right)\left(2\right)\ldots \left(8i-8\right)!. \end{array}\right.$$

Lemma 15: Let i be the number of chains. Then, the number of Ω -nested Abacus in the generating tree by adding a beta number in chain i at level n is

$$\left\{ \begin{array}{c} L_{O}=1\;\\ L_{1}=8i-8\\ L_{2}=8i-7\\ L_{3}=8i-6\\ \vdots \\ \vdots \\ L_{n}=\left(8i-1\right)!. \end{array}\right...\ldots \ldots \ldots \left(*\right)$$

## Generating Function (G.F)

Theorem. Let

In order to define G.F., first we have to find a succession rule, ϑ . Which is

$$\vartheta =\left\{ \begin{array}{ccc} \left(1\right) & & \\ \left(8i-8\right) & \to & \left(1\right)\left(2\right)\ldots \left(8i-8\right)!. \end{array}\right.$$

Then we must find the function. $g_{n}\left(x,y\right)$ and $g_{n}\left(x\right)$ when y=1 to find the generating function. We can define a sequence of positive integers. $g_{n},n\;\geq \;0$ , where $g_{n}$ The number of nodes at the general level (level n). In the succession-rule, ϑ (*), all nodes are changed to (8i-8). Then:

$$g_{n}\left(x,y\right)=\sum_{\begin{array}{c} n\;\geq \;0\\ i\;\geq \;0 \end{array}}g_{n,8\left(i-1\right)}$$

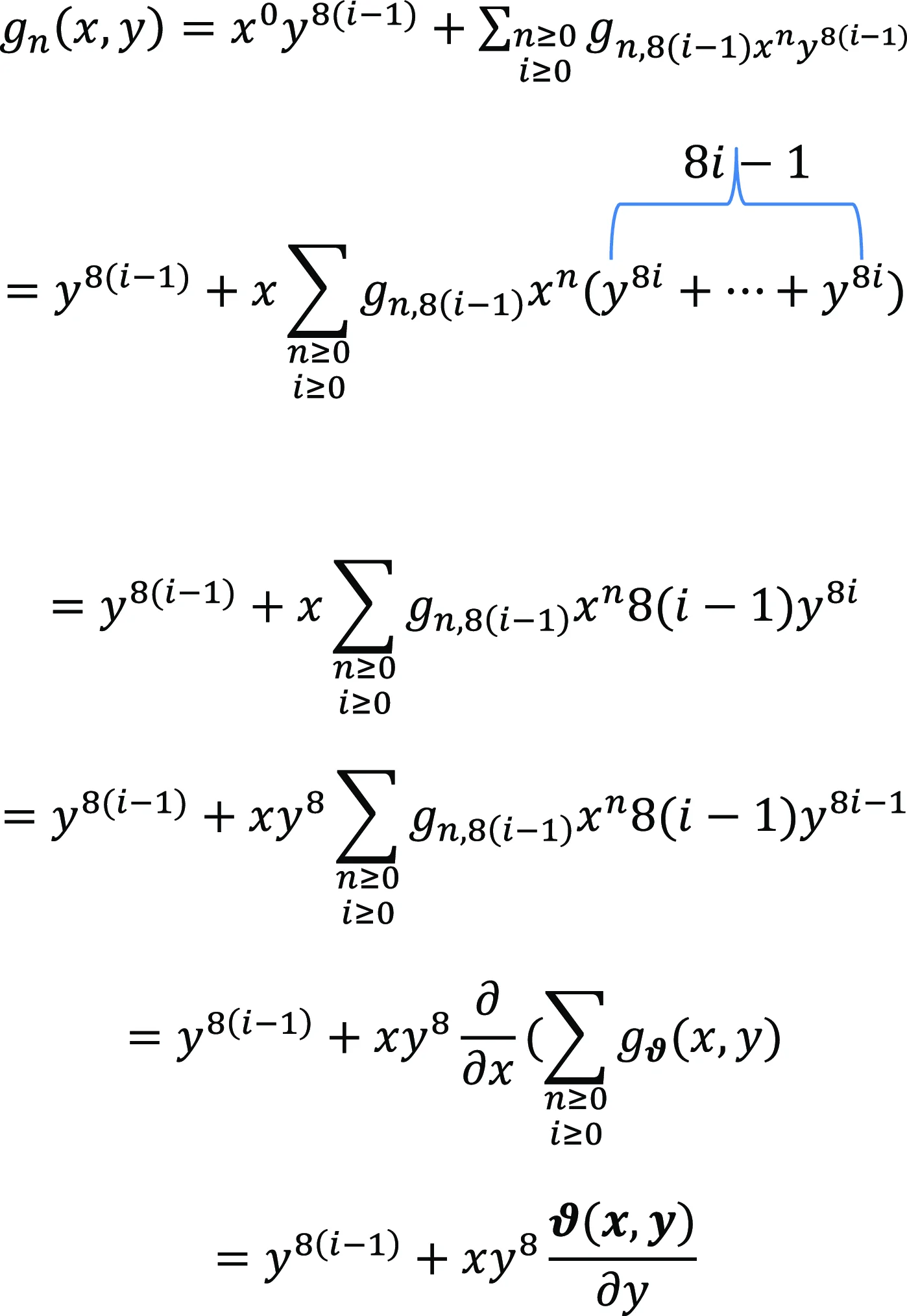

$$\frac{\partial g_{\vartheta }\left(x,y\right)}{\partial y}-\frac{1}{xy^{8}}g_{\vartheta }\left(x,y\right)=\frac{-y^{8i-8}-8}{x}$$

$$\begin{array}{c} g_{\vartheta }\left(x,y\right)=e^{\int -xy^{8}\mathrm{dy}}\left(c+\int \frac{-y^{8i-8}-8}{x}\;e^{\int \frac{1}{xy^{8}}\mathrm{dy}}\mathrm{dy}\right)\\ =\frac{1}{e^{7}xy^{7}}\left(c-\frac{\int y^{8\left(i-1\right)}e^{\frac{1}{-7xy^{7}}\mathrm{dy}}}{x}\right) \end{array}$$

$$\lim_{x⟼0}g_{\vartheta }\left(x,1\right)=\lim_{x⟼0}\;\frac{1}{e^{7}x}\left(c-\lim_{x⟼0}\frac{e^{\frac{1}{-7x}}}{x}\right)$$

$$e^{8}\left(c-0\right)=0$$

Then c=0

$$g_{\vartheta }\left(x,y\right)=\frac{1}{e^{7}xy^{7}}\left(-\frac{\int y^{8i}e^{\frac{1}{-7xy^{7}}\mathrm{dy}}}{x}\right)$$

## Conclusion

This study examines a class of polyominoes known as the Ω-nested Abacus. Initially, we study the characterised of a new class by specific geometric constraints defined by sets of rows, columns, and chains. A series of operations on polyominoes is then introduced using a partition-theoretic construct known as the beta number. Furthermore, a set of operations on polyominoes is developed using a partition-theoretic construct known as the beta number, enabling localised transformations of the objects. Furthermore, A succession rule is formulated based on tree structures that illustrate the developmental patterns of these trees. A recursive method is established for the systematic generation of these objects of a given size through the use of generating trees.

## Ethical approval

Ethical approval was not required for this study.

## Data Availability

No data availability with this atrial as the study is purely theoretical and does not involve the generating analysis or use of any datasets.
