# Enumeration Class of Polyominoes Inscribed in James Abacus and Related ECO

**DOI:** 10.12688/f1000research.172910.2

**Published:** 2026-05-15

**Authors:** Eman F. Mohommed, jalal Abd Jassim

**Affiliations:** 1 mathematic, Mustansiriyah University Department of Mathematics, Baghdad, Baghdad Governorate, Iraq

**Keywords:** Polyominoes, Class, Enumeration, Succession-rule, Abacus diagram

## Abstract

This paper studies a class of polyominoes. The new class is defined through a representation on the James abacus through nested chain which denoted Ω -nested Abacus. Depended on partition, beta number, nested chain we defined the structural condition this class. A local transformations, called SSPT-transformation and MSPT-transformation are formulated on the even chains. Then, we drive explicit formulas for the number of position in each chain, number of even chain and the total number of all chains. The structure of new class lead to enumeration formulas for the objects produced after application new transformation. To enhance further, based on these classes, generating functions are also being formulated by employing enumeration of combinatorial objects (ECO). In ECO method, each object is obtained from smaller object by making some local expansions. These local expansions are described in a simple way by a succession rule which can be translated into a function equation for the generating function.

## Introduction

**Figure 1.**
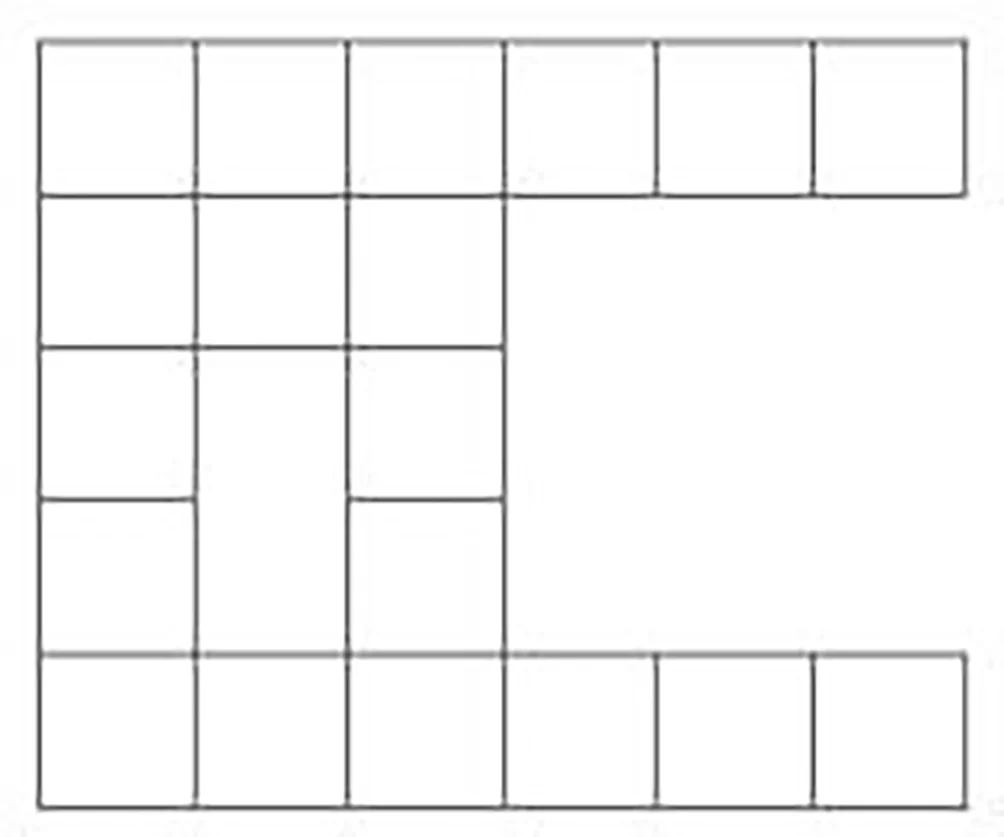
22-polyominoes (polyominoes with 22 connected ominoes).

Polyominoes are finite configurations composed of unit squares, known as ominoes, that are joined edge to edge to form a connected interior, as illustrated in Figure 1.

**Figure 2.**
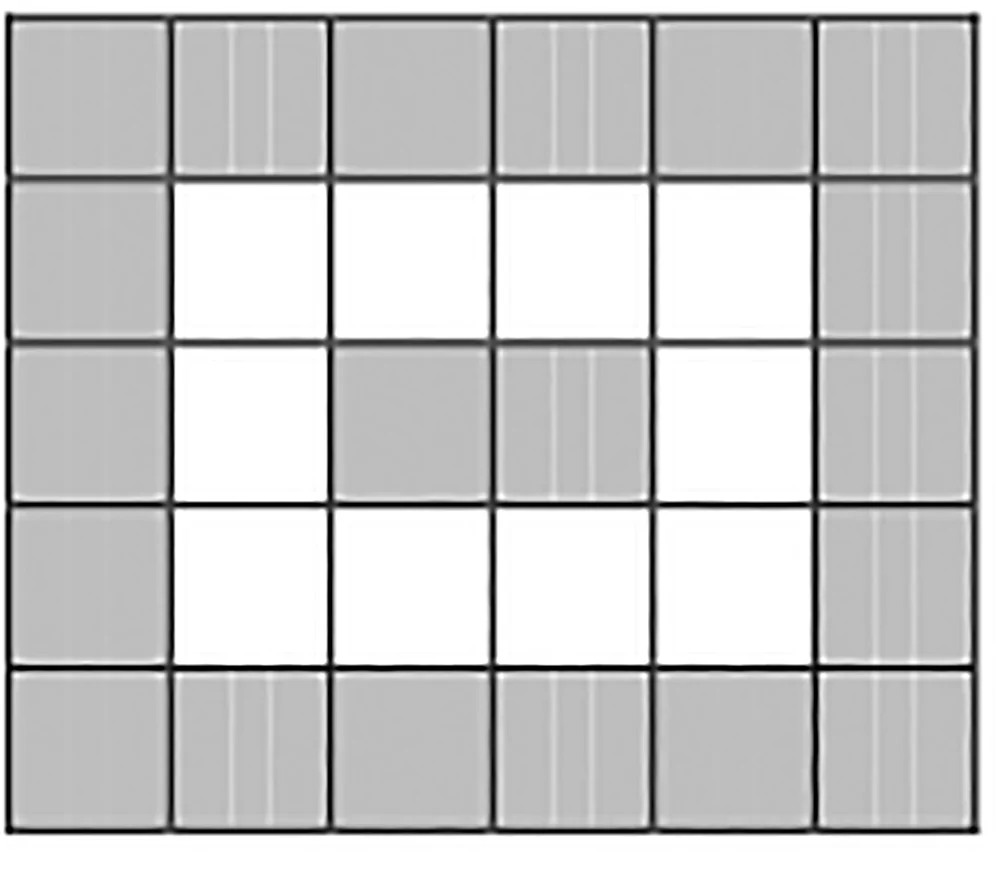
Nested chain abacus with 6 columns, 5 rows and 3 chains.

An n -polyomino (a polyomino consisting of n ominoes) is defined up to translation, and the concept is commonly attributed to Golomb. ^1^ In contemporary research, *n*-polyominoes have attracted significant attention from computer scientists, physicists, mathematicians, and biologists. Despite extensive studies, enumerating n-polyominoes remains a challenging and unresolved problem in combinatorial geometry, representing one of its most fundamental open questions. ^2– 5^ There is no closed-form equation for *n*-polyominoes, and the problem has been solved up to *n* < 56. ^3^ No closed-form expression for the enumeration of n -polyominoes is currently known. Due to the complexity of this problem, several simpler subclasses of n -polyominoes have been formulated and extensively examined in the literature. ^6– 8^ E.F. provide a new representation of *n*-polyominoes (Plyominoes with *n* ominoe) using one of the graphical representations of partition (James-diagram) called nested chain abacus (N.C.A.) with b -columns and d -rows. ^9^ The Nested Chain Abacus (N.C.A.) offers a novel framework for representing any n connected omino, including empty ones (holes), through the use of a beta number. In this new representation, n -polyominoes are organised into a series of nested chains. ^10^ A Nested Chain Abacus (N.C.A.) is an n -polyomino inscribed within a James diagram, consisting of both outer and inner chains. The inner chains are numbered from 1 to n , where n is a positive integer, and chain 1 is the innermost chain. No intersections occur among any of the chains. Through this new representation, and for the first time, each *n*-polyomino has been systematically associated with a unique code. Next Figure 2 illustrates an example of an N.C.A. with six columns, five rows, and three chains.

## Terminologies and definition

This section introduced some importuned definitions:

Definition 1. ^4^A partition of a positive integer, k , is a sequence of integers $\lambda_{1},\lambda_{2},\ldots ,\lambda_{n}$ such that $\lambda_{1}\;\geq \;\lambda_{2}\;\geq \;\ldots \;\geq \;\lambda_{n}$ and $\sum_{i=1}^{n}\lambda_{i}=k$ .

Example 1.
λ=(6,6,3,2,1,1) is a partition of 19

Definition 2. ^4^A sequence of positive numbers { $\;\beta_{1},\beta_{2},\ldots ,\beta_{k}\}\;$ called beta number such that $\;\beta_{i}=\lambda_{i}+k-i\;$ and 1≤i≤k .

Example 2. ^4^Consider of Example 1, λ=(6,6,3,2,1,1) then beta number of λ is

$$\;\beta_{i}=\lambda_{i}+b-i,\;\text{where}\;i=1,2,3,4,5,6$$

And hence

$$\;\beta_{1}=11,\;\beta_{2}=10,\;\beta_{3}=6,\;\beta_{4}=4,\;\beta_{5}=2,\;\beta_{6}=1$$

Thus beta number sequence is

{11,10,6,4,2,1}.

Definition 3. ^4^James Abacus is a graphical representation of a partition of any integer number using beta numbers.

**Figure 3.**
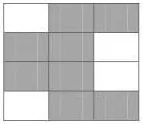
James’s Abacus with 3 columns, 4 rows and 6 beta.

Consider Example 1, the James Abacus with 1,2,3,4,6,7,10,11 beta position as show in next Figure 3.

Definintion 4. ^9^A new representation of *n*-polyominoes (Plyominoes with *n* ominoe) using one of the graphical representations of partition (James-diagram) called nested chain abacus (N.C.A.) with *b*-columns and *d*-rows.

**Figure 4.**
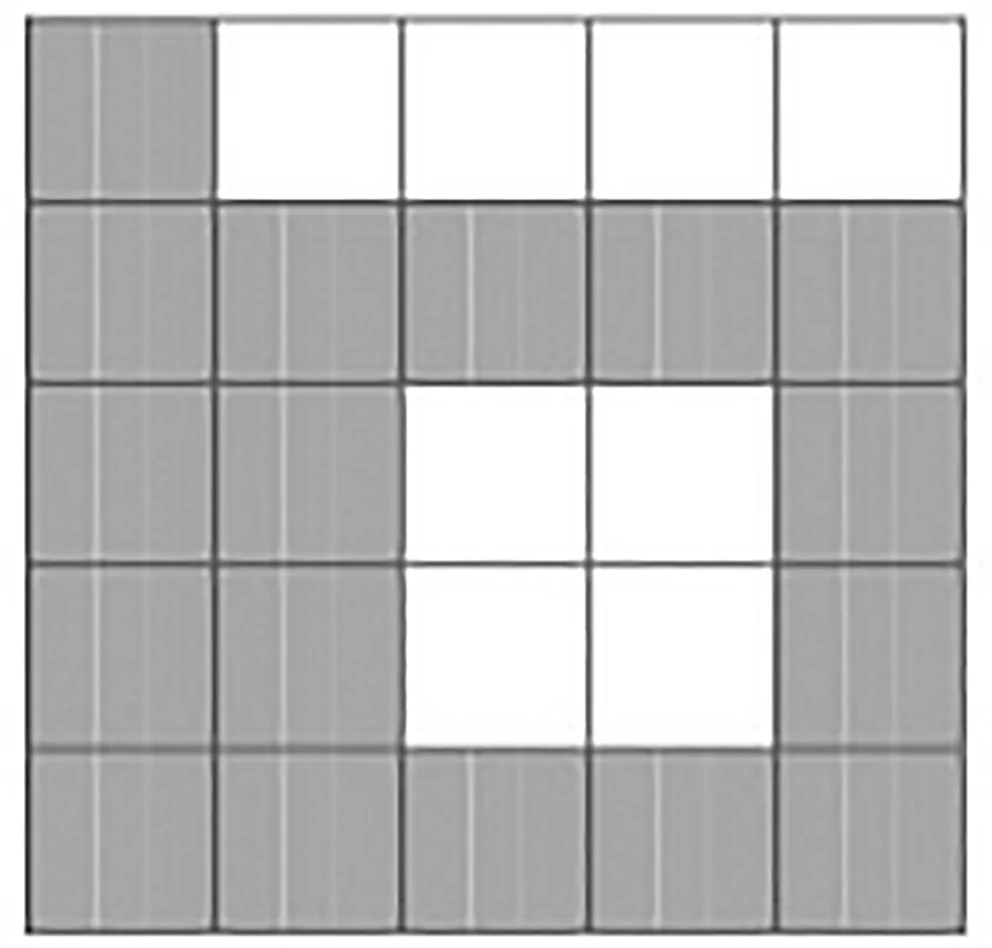
N.C.A. with 5 columns ( b=5 ) and 3 chains represented of 94, where the beta number sequence is { 0,5,6,7,8,9,10,11,14,15,16,19,20,21,24}.

Next Figure 4 gives an example of N.C.A. with 3 chains represented of 94, where the beta number sequence is {0,5,6,7,8,9,10,11,14,15,16,19,20,21,24}.

Definition 5: A connected chain is a chain with only beta positions.

**Figure 5.**
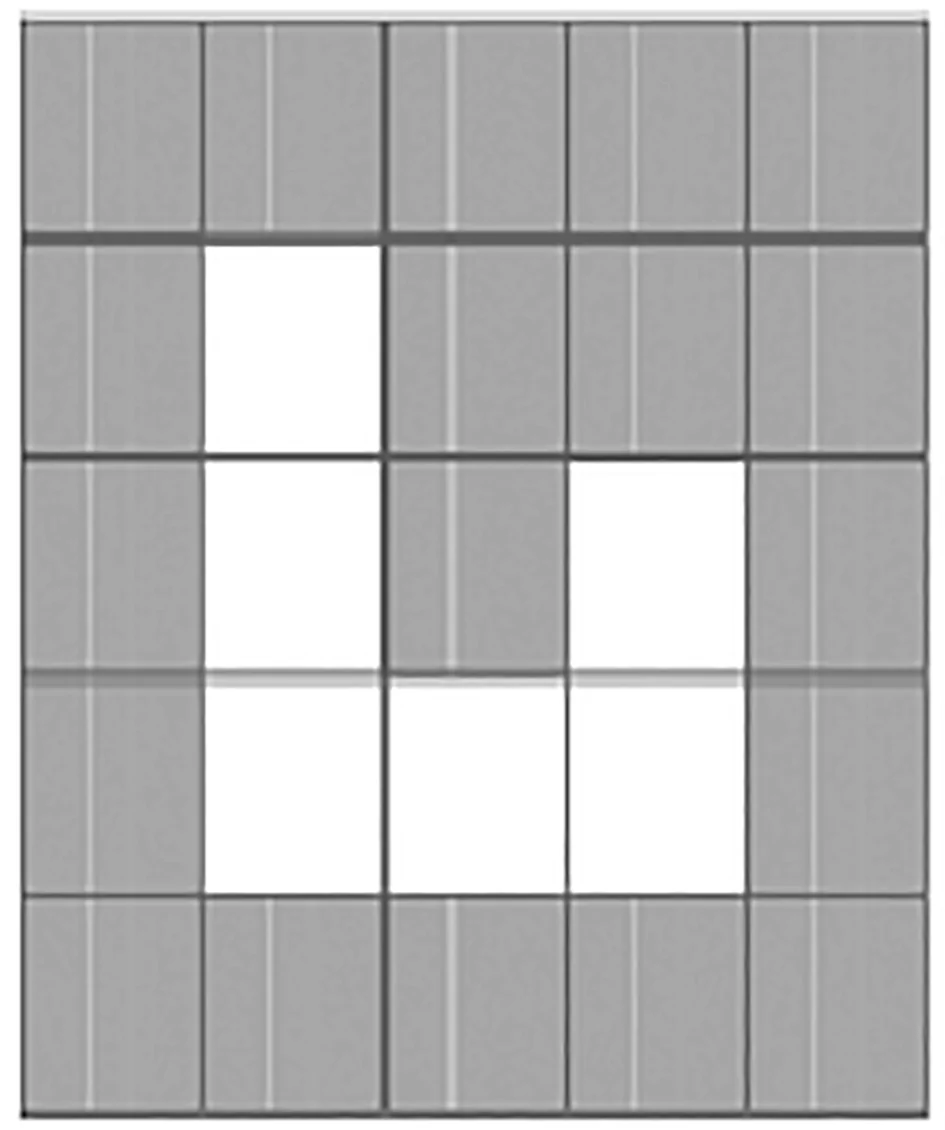
N.C.A. with 1 connected chains (chain 3).

Next Figure 5 gives an example of N.C.A. represented by a beta sequence {0,1,2,3,4,5,7,8,9,10,12,14,15,19,20,21,22,23,24} with one connected chain (chain 1).

Definition 6. Let $\;\beta_{i},\;\beta_{j}$ be two beta numbers in N.C.A. then $\;\beta_{i},\;\beta_{j}$ are connected iff

1-
$\left|\;\beta_{i}-\beta_{j}\;\right|=1$ if $\;\beta_{i},\;\beta_{j}$ Located in the same row.
2-
$\left|\;\beta_{i}-\beta_{j}\;\right|=b\;\text{if}\;\;\beta_{i},\;\beta_{j}\;\text{Located in the same column}$ .

For example, the beta set

{0,5,6,7,8,9,10,11,14,15,16,19,20,21,24}.

From Figure 4 is a connected.

Definition 7. Each nested chain abacus (N.C.A.) with b columns and *b* rows is called a polyamines if every two beta numbers are connected.

Definition 8. Let N.C.A. be a nested chain abacus consist of b column and d rows, and let $C_{1},C_{2},\ldots ,C_{r}$ be its chains, order from the innermost chain to the outermost chain. The NCA is called an Ω -nested Abacus if the following condition are satisfied

1- First chain (initial chain) $\;C_{1}$ which consist exactly one position.
  a. If $C_{1}$ consist exactly one beta position, then $C_{2}$ has at least five beta positions.
  b. If the position in $C_{1}$ is empty position, then $C_{2}$ consist k beta positions, where 1≤k≤8

2- Every even chain ( $C_{2t})$ may contain both beta position and empty beta position.
3- Every odd chain ( $C_{2t-1})$ forms a connected chain.

Any polyomino represented by such a configuration is called an Ω -nested Abacus polyomino.

**Figure 6.**
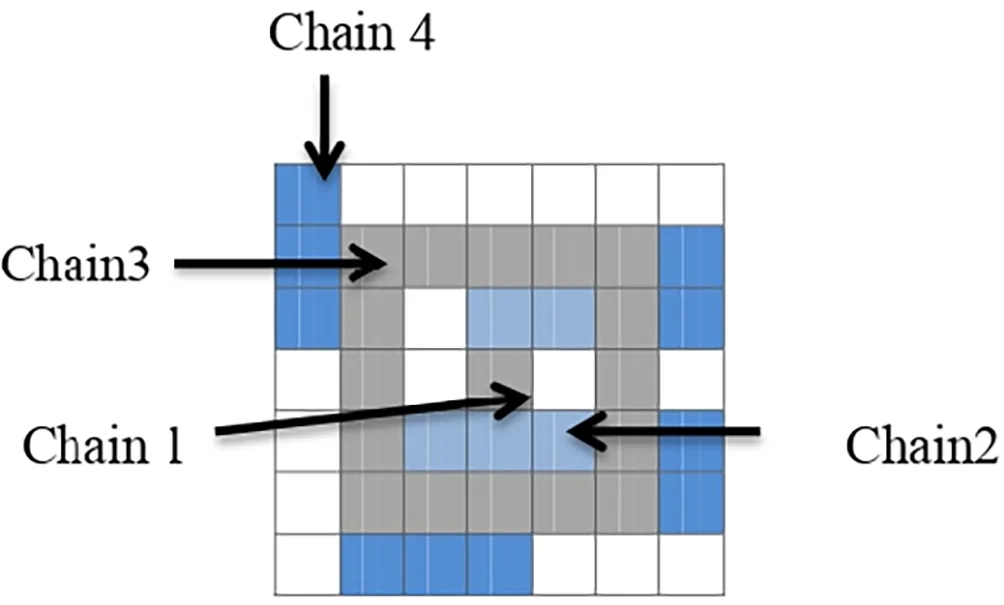
Ω -nested Abacus with four chains.

Figure 6 gives an example of Ω -nested Abacus with four chains, where

Not Every polyamines inscribed in a nested chain-abacus is Ω -nested Abacus.

Figure 4 given an example of N.C.A. but not Ω -nested Abacus, while Figure 6 given an example of Ω -nested Abacus.

Not. Throughout our result the underlying new family ( Ω -nested Abacus) assumed b=d where b is odd number. Since every odd chain is completely filled, so only even chain well be contribute to the generating process.

The algorithm construct to generate classes of new family depended on the following transformation.

Definition 9. Single – beta transformation (SPT)

Let an Ω -nested Abacus with b column, d rows and $C_{i}$ chains. Suppose that a beta number β=(m−1)b+(j−1) in chain $C_{i}$ is located in row m and column j such that 1≤m≤d , 1≤j≤b and 1≤i≤r . A single beta transformation (SPT) in chain $C_{i}$ is local transformation ( $\beta \to \overset{`}{\beta }$ ) within the same chain where

$$\overset{`}{\beta }=\{\begin{array}{ccc} \left(m-1)b+(j-2\right) & \text{if} & i+1\;\leq \;m\;\leq \;(r-i+1),\;j=b-i+1\\ \mathrm{mb}+(j-1) & \text{if} & i\;\leq \;m\;\leq \;(r-i),\;j=i\\ (m-1)b+j\; & \text{if} & m=i,\;i+1<j\;\leq \;b-i+1\\ \left(m-2)b+(j-1\right) & \text{if} & m=d-i+1,\;i\;\leq \;j<b-i. \end{array}$$

Remark 10. The maximal number of transformations in chain n is equal to the number of positions in the chain, where n>1 .

**Figure 7.**
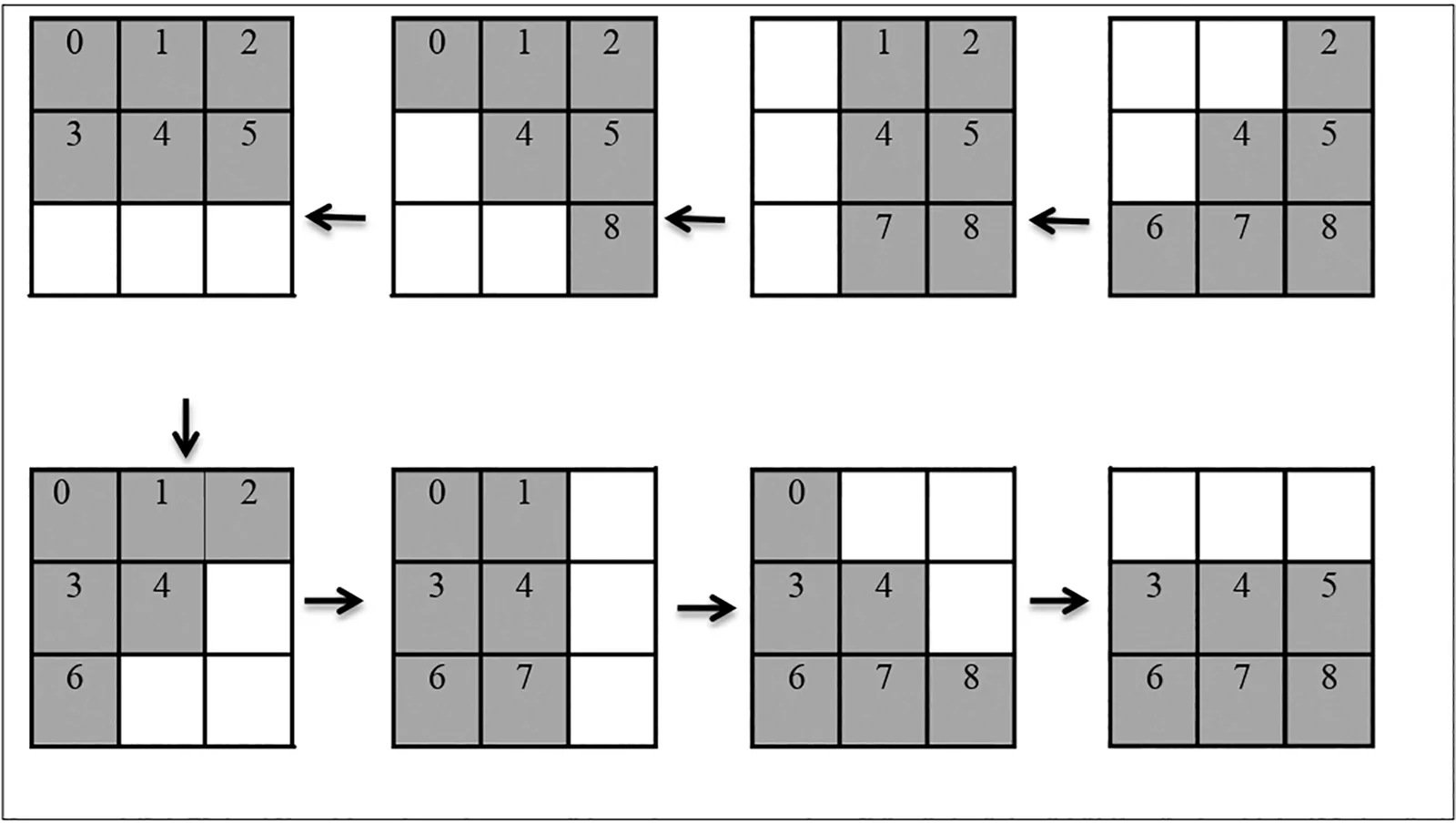
Illustrates Single – beta transformation (SPT) on Ω -nested Abacus.

Figure 7 illustrates Single – beta transformation (SPT)

Not.

1- SSpt is a SPT transformation in one chain.
2- MSpt is a SPT transformation in all chains.

## Enumeration of the Ω -nested Abacus class

Next, the enumeration of Ω-nested Abacus with *b* columns and *d* rows is presented.

Lemma 11. Let an Ω -nested Abacus with b column, d rows and $C_{r}$ chains then, the number of position in any chains is $|C_{i}|=8(n-1).$

Proof. Based on Definition 9 $C_{1}$ consists of a single position. Then, the successive chain form a discrete ring around the single position with four side increments and four corner position. Then $|C_{2}|=8$ , thus $|C_{n}|=|C_{n-1}|+8$ where 1≤n≤r . Thus

$$\left|C_{n}|=8(n-1\right)$$

Where n>1 .

Corollary 12. Based on Remark 10 and Lemma 11, the number of (SSPT) transformation in chain $C_{n}$ is equal to 8(n−1) .

Lemma 13. Let Ω-nested Abacus be a polyomino intercept in N.C.A. with b columns and d rows such that b=d and b is odd. Then the total number of chain is $\frac{b+1}{2}\;$ .

Proof. Based on Ω-nested Abacus structure is symmetric with respect to a central column (central chain $C_{n}$ ) then for all *i*-th chains, the boundary columns must satisfy i≤b−i+1 , thus

$2i\;\leq \;b-1\;\to \;i\;\leq \;\frac{b+1}{2}$ . Then every Ω-nested Abacus with $\frac{b+1}{2}$ chains.

Proposition 14. Let Ω-nested Abacus be a polyomino intercept in NCA with b columns and d rows such that b=d and b is odd. Then the number of even chain is $\frac{b-1}{4}$ .

Provided that b≡1(mod4).

Proof. Based on Lemma 13 the number of chain in Ω-nested Abacus is $t=\frac{b+1}{2}$ . The even chains among 1,2,…,t are $2,4,\ldots ,\lfloor \frac{t}{2}\rfloor$ . Since $t=\frac{b+1}{2}$ is even then $\frac{t}{2}=\frac{\frac{b+1}{2}}{2}$ is even since the first chain is odd and fixed. Then there are Since $t=\frac{b+1}{2}$ is even then $\frac{t}{2}=\frac{\frac{b+1}{2}-1}{2}=\frac{b-1}{4}$ , then the number of even chain is $\frac{b-1}{4}$ .

Next we found the number of Ω-nested Abacus if we application SSPT-Transformation in chain i .

Lemma 15: Let Ω-nested Abacus be a polyomino intercept in NCA with b columns and d rows such that b=d , b is odd and let n be even number. Then the number of Ω-nested Abacus generating by employ SSPT-Transformation exactly chain n is 8(n−1).

Proof. By Lemma 11 the number of admissible positions in chains n is 8(n−1) . Since move each beta position yields a distinct Ω-nested Abacus under SSPT-transformation obtain in chain n with 8(n−1) position is 8(n−1) .

Theorem 16: Let Ω-nested Abacus be a polyomino intercept in N.C.A. with b columns and d rows such that b=d , b is odd and let n be even number. Then the number of Ω-nested Abacus generating by employ SSPT-Transformation is

∑∀n(R(n−1)8)

Where $R=\sum_{n}^{\left\lfloor \frac{b+1}{4}\right\rfloor }(n-1)8$ , b≡1(mod4)

Proof. Based on Lemma 10, the number of positions in chain *n* is $(n-1)2^{3}$ by Lemma 11 and Definition 8, there are $\frac{\;b-1}{4\;}$ chain with beta and empty beta position in Ω-nested Abacus. The maximal number of transformations in chain n is equal to ((n−1)8) . Since each move generates a new Abacus (polyominoes), thus there are ((n−1)8) of Ω-nested Abacus generated by employing SSPt transformation. As a result of this, there are

∑∀n(R(n−1)8)

Ω-nested Abacus.

Theorem 17. Let Ω-nested Abacus be a polyomino intercept in N.C.A. with b columns and d rows such that b=d , b is odd and let n be even number. Then the number of Ω-nested Abacus generating by employ SSPT-Transformation exactly one even chain.

$$\sum_{n=2}^{\left\lfloor \frac{b+1}{4}\right\rfloor }8(n-1).$$

Where b≡1(mod4)

Proof. Based on Lemma 11 the number of positions in chain n is 8( *n*−1), any position in the chain will be determines one distinct SSPT in that chain. Thus all even chain $C_{2},C_{4},\ldots ,$ gives $\sum_{n=2}^{\left\lfloor \frac{b+1}{4}\right\rfloor }8(n-1)$ .

Theorem 18. Let Ω-nested Abacus be a polyomino intercept in N.C.A. with b columns and d rows such that b=d , b is odd and let n be even number. Then the number of Ω-nested Abacus generating by employ MSPT-Transformation exactly chain n is

$$\prod_{\forall n}8(n-1)$$

Where b≡1(mod4) and $n=2,4,\ldots ..,\frac{b=1}{4}$

Proof. Based on Lemma 11, the number of admissible beta and empty eta position in chain *n* is 8(n−1) . Since the odd chain are completely filled, the remain fixed and do not contribute to the transformation. By Lemma 15 the number of Ω-nested Abacus generating by employ SSPT-Transformation exactly chain n is 8(n−1). Since the MSPT-transformation acts simultaneous on all even chain. A generated Ω-nested Abacus is obtain by selecting one admissible position in each chain. The choice in one even chain does not restrict the admissible choices in any other even chain. Then the number of Ω-nested Abacus can be generated after application SSPT-Transformation is ∏∀n8(n−1) .

## Generating function with respect to chains

In this section, the method described in ^11, 12^ is employed to enumerate the Ω-nested Abacus class, representing polyominoes inscribed within a James diagram. The ECO (Enumerating Combinatorial Objects) method has previously been applied to the enumeration of various polyomino classes. ^12^ This approach is based on a *succession rule.*

### Generating Function (G.F.)

Let $C_{2k}$ be even chain of Ω-nested Abacus, based on Lemma 11 the number of positions in any chain is

$$M_{k}=\lceil \;C_{2k}\rceil =8(n-1).$$

**Figure 8.**
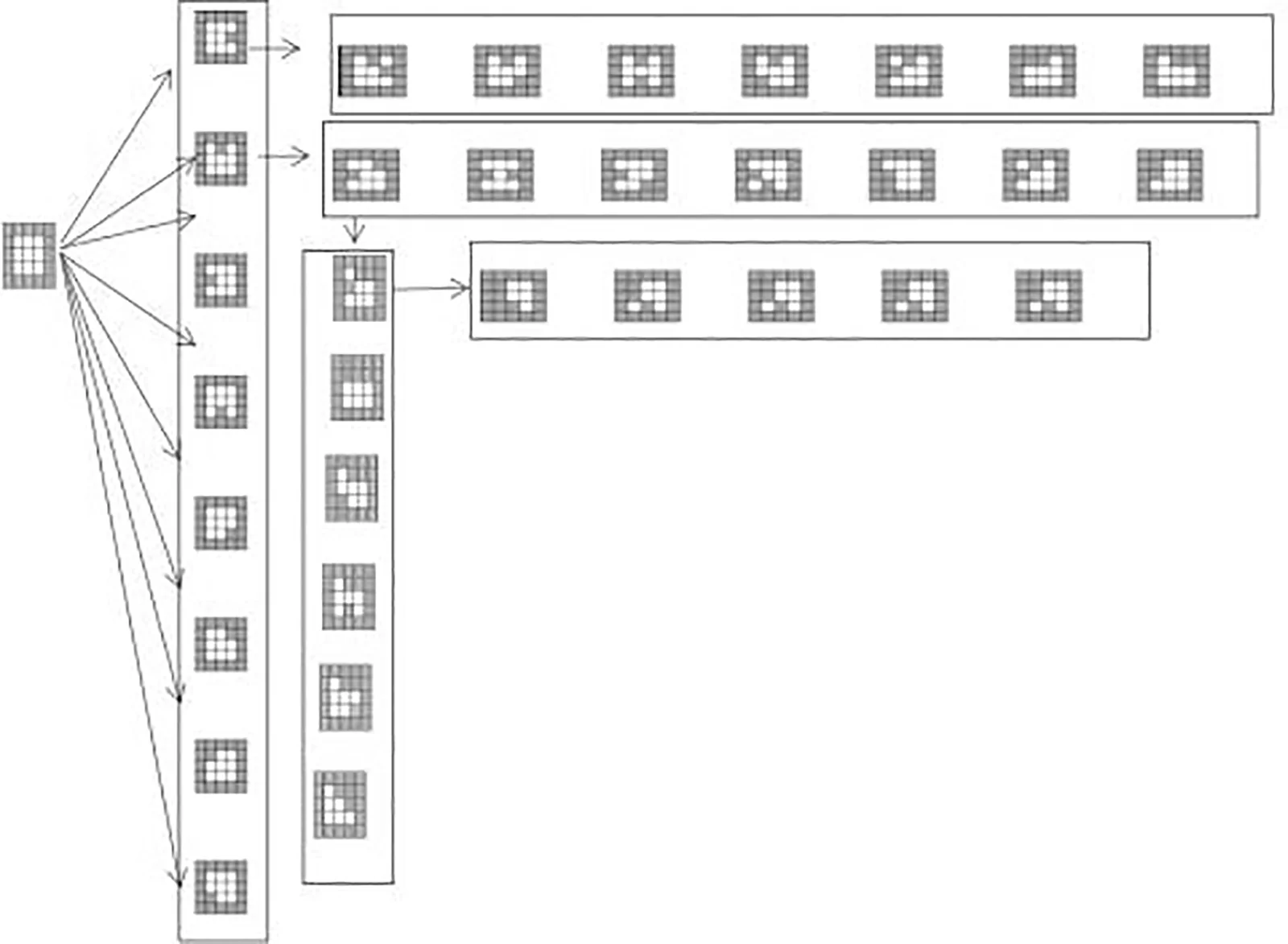
First and second level of ϑ using Ω -nested Abacus class with 5 columns, 5 rows and 3 chain.

We application SSPT-Transformation inside this chains such that no previously select position may be chose again. Thus, in initial stage there are $M_{k}$ choices ( $M_{k}$ of Ω-nested Abacus), after one insertion we have only $M_{k}-1$ position ( $M_{k}-1$ of Ω-nested Abacus), after two distinct insertion there are only $M_{k}-2$ position ( $M_{k}-2$ of Ω-nested Abacus as shown in Figure 8), and so on. Thus, there are

$$a_{n}^{k}=M_{k}(M_{k}-1)(M_{k}-2)\ldots (M_{k}-n+1),2\;\leq \;n\;\leq \;M_{k}$$

Above process is encoded by a generating tree. The root labeled by $M_{k}$ , each node will be produces j children, every one of j children will be product j−1 . Thus the local succession rule is

$$\vartheta =\{\begin{array}{ccc} M_{k} & & \\ j & \to & {\left(j-1\right)}^{j}.1\;\leq \;j\;\leq \;M_{k} \end{array}$$

Assume that $a_{n+1}^{k}$ denoted the number of nodes at level n ϑ yield the recurrence

$$a_{n+1}^{k}=(M_{k}-n)a_{n}^{k}$$

Where $a_{0}^{k}=1,2\;\leq \;n\;\leq \;M_{k}$ .

$$a_{n}^{k}=\frac{M_{k}!}{(M_{k}-n)!}.$$

Using the ordinary level-generating polynomial of the even chain

$$C_{k}(x)=\sum_{n=2}^{M_{k}}(M_{k}{)}_{n}x^{n}$$

$$C_{k}(x)=\sum_{n=2}^{M_{k}}\frac{{\left(M_{k}\right)}_{n}}{n!}x^{n}$$

Since

$$\frac{{\left(M_{k}\right)}_{n}}{n!}=\frac{M_{k}!}{(M_{k}-n)!n!}=(\begin{array}{c} M_{k}\\ n \end{array})$$

then,

$$C_{k}(x)=\sum_{n=2}^{M_{k}}(\begin{array}{c} M_{k}\\ n \end{array})x^{n}=(1+x{)}^{M_{k}}$$

$$C_{k}(x)=\sum_{n=2}^{M_{k}}(\begin{array}{c} M_{k}\\ n \end{array})x^{n}=(1+x{)}^{8(2k-1)}$$

Then the generating function

$$C_{k}(z,x)=\sum_{k=2}^{m}z^{k}(C_{k}(x))=\sum_{n=2}^{M_{k}}z^{k}(1+x{)}^{8(2k-1)}$$

Where $m=\lfloor \frac{b+1}{4}\rfloor$

Thus

$$C_{k}(z,x)=\sum_{n=2}^{M_{k}}(z(1+x{)}^{16}{)}^{k}$$

$$C_{k}(z,x)=z(1+x{)}^{8}\frac{1-(z(1+x{)}^{16}{)}^{m}}{1-z(1+x{)}^{16}}$$

$C_{k}(z,x)$ is generating function to enumerated the number of Ω-nested Abacus.

## Conclusion

This study examines a class of polyominoes known as the Ω-nested Abacus. Initially, we provide a characterization of a new class based on specific geometric constraints defined by rows, columns, and chains. A series of operations on polyominoes is then introduced using a partition-theoretic construct known as the beta number. Furthermore, a set of operations on polyominoes is developed using a partition-theoretic construct known as the beta number, allowing for localized transformation of the structure. Furthermore, A succession rule is formulated based on generating tree techniques to describe the growth of these object. Based on this framework a recursive method is established for the systematic generation of Ω-nested Abacus configuration of a given size through the use of generating trees.

## Ethical approval

Ethical approval was not required for this study.

## Data Availability

No data availability with this atrial as the study is purely theoretical and does not involve the generating analysis or use of any datasets.
