# Enumeration Class of Polyominoes Inscribed in James Abacus and Related ECO

**DOI:** 10.12688/f1000research.172910.3

**Published:** 2026-07-30

**Authors:** Eman F. Mohommed, jalal Abd Jassim

**Affiliations:** 1 mathematic, Mustansiriyah University Department of Mathematics, Baghdad, Baghdad Governorate, Iraq

**Keywords:** Polyominoes, Class, Enumeration, Succession-rule, Abacus diagram

## Abstract

This paper studies a class of polyominoes. The new class is defined through a representation on the James abacus through nested chain which denoted Ω -nested Abacus. Depended on partition, beta number, nested chain we defined the structural condition this class. A local transformations, called SSPT-transformation and MSPT-transformation are formulated on the even chains. Then, we drive explicit formulas for the number of position in each chain, number of even chain and the total number of all chains. The structure of new class lead to enumeration formulas for the objects produced after application new transformation. To enhance further, based on these classes, generating functions are also being formulated by employing enumeration of combinatorial objects (ECO). In ECO method, each object is obtained from smaller object by making some local expansions. These local expansions are described in a simple way by a succession rule which can be translated into a function equation for the generating function.

## Introduction

Polyominoes are finite configurations composed of unit squares, known as ominoes, that are joined edge to edge to form a connected interior, as illustrated in Figure 1.

**Figure 1.**
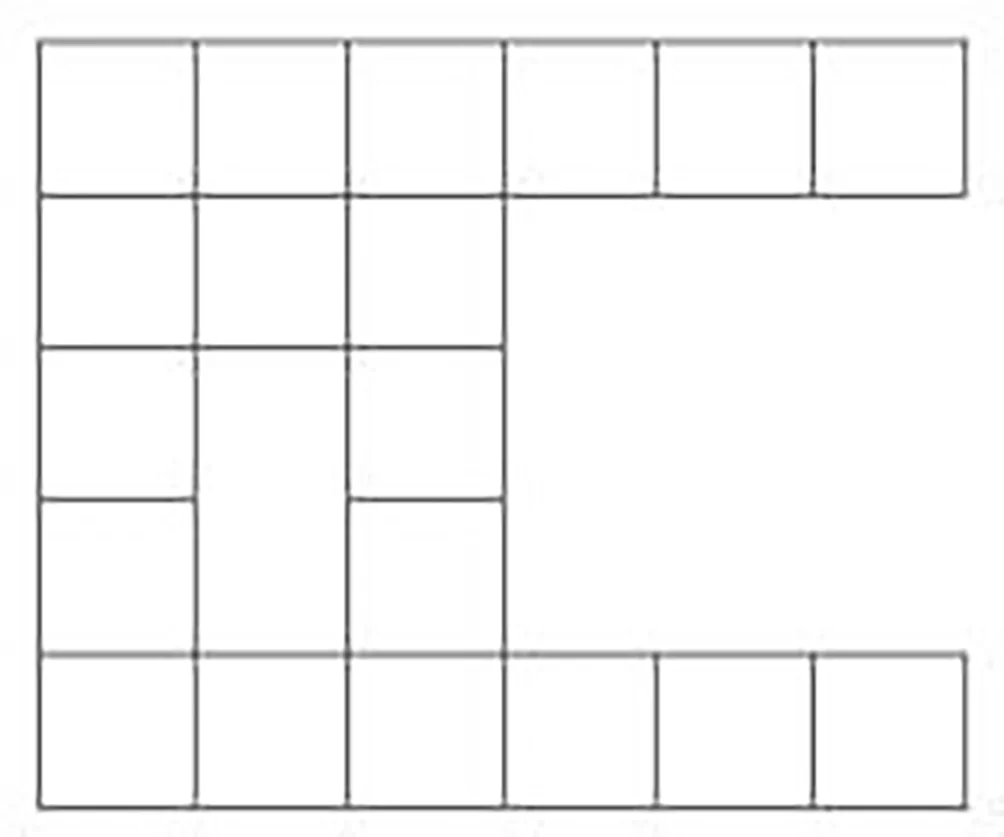
22-polyominoes (polyominoes with 22 connected ominoes).

An n -polyomino (a polyomino consisting of n ominoes) is defined up to translation, and the concept is commonly attributed to Golomb. ^1^ In contemporary research, *n*-polyominoes have attracted significant attention from computer scientists, physicists, mathematicians, and biologists. Despite extensive studies, enumerating n-polyominoes remains a challenging and unresolved problem in combinatorial geometry, representing one of its most fundamental open questions. ^2– 5^ There is no closed-form equation for *n*-polyominoes, and the problem has been solved up to *n* < 56. ^3^ No closed-form expression for the enumeration of n -polyominoes is currently known. Due to the complexity of this problem, several simpler subclasses of n -polyominoes have been formulated and extensively examined in the literature. ^6– 8^ E.F. provide a new representation of *n*-polyominoes (Plyominoes with *n* ominoe) using one of the graphical representations of partition (James-diagram) called nested chain abacus (N.C.A.) with b -columns and d -rows. ^9^ The Nested Chain Abacus (N.C.A.) offers a novel framework for representing any n connected omino, including empty ones (holes), through the use of a beta number. In this new representation, n -polyominoes are organised into a series of nested chains. ^10^ A Nested Chain Abacus (N.C.A.) is an n -polyomino inscribed within a James diagram, consisting of both outer and inner chains. The inner chains are numbered from 1 to n , where n is a positive integer, and chain 1 is the innermost chain. No intersections occur among any of the chains. Through this new representation, and for the first time, each *n*-polyomino has been systematically associated with a unique code. Next Figure 2 illustrates an example of an N.C.A. with six columns, five rows, and three chains.

**Figure 2.**
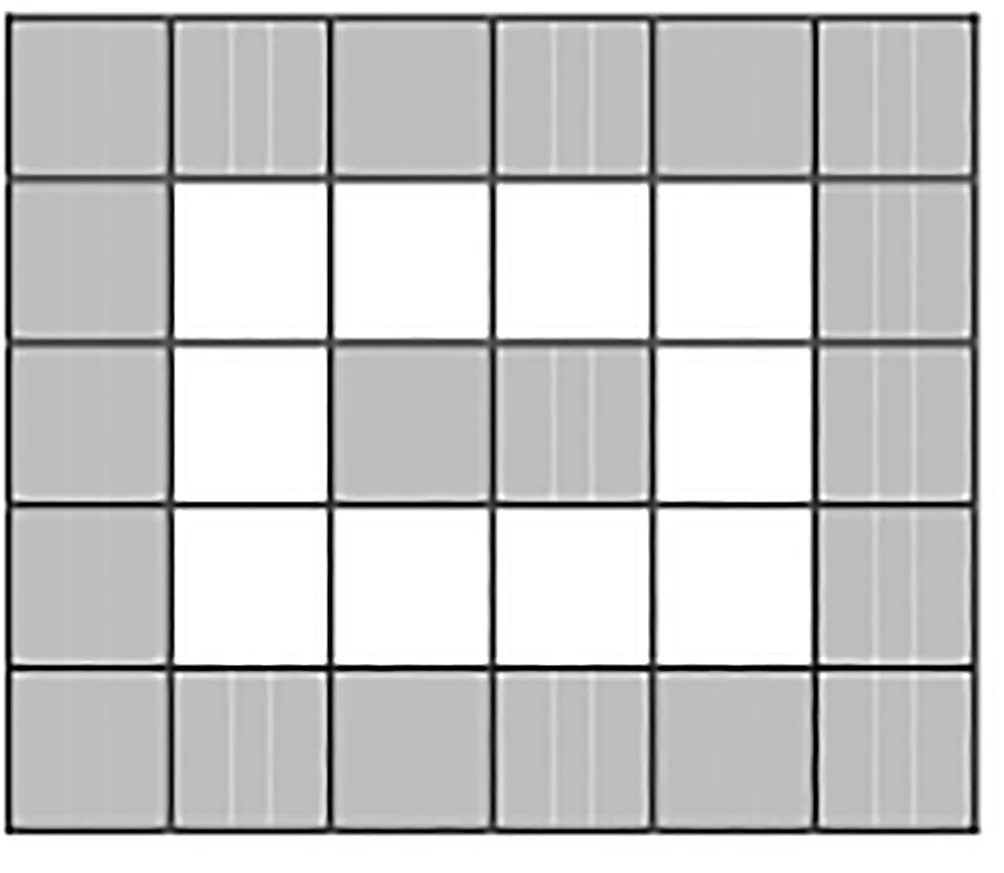
Nested chain abacus with 6 columns, 5 rows and 3 chains.

Although the nested chain abacus provides a general representation pf polyominoes using the James Abacus provides a general representation of polyominoes using the James Abacus, its general structure is not specifically designed for systematic combination enumeration through local transformation. In particular, the absence of structural restriction on the arrangement of chains makes it difficult to derive explicit counting formulas and generating function. This limitation properties required for transformation-based enumeration.

To address this issue, we introduce the Ω-nested Abacus, a restricted family of Nested chain Abacus representations characterized by specific conditions imposed on the odd and even chains. These conditions enable the definition of local transformation and provide a convenient framework for deriving explicit enumeration formulas using ECO methodology.

## Terminologies and definition

This section introduced some importuned definitions:

Definition 1. ^13^A partition of a positive integer, k , is a sequence of integers $\lambda_{1},\lambda_{2},\ldots ,\lambda_{n}$ such that $\lambda_{1}\;\geq \;\lambda_{2}\;\geq \;\ldots \;\geq \;\lambda_{n}$ and $\sum_{i=1}^{n}\lambda_{i}=k$ .

Example 1.
λ=(6,6,3,2,1,1) is a partition of 19

Definition 2. ^13^ Let $\lambda =\;(\lambda_{1},\lambda_{2},\ldots ,\lambda_{n})\;$ be a partition of a positive integer ( *k*) where ( *n*) denotes the number of parts of the partition. The corresponding beta number sequence is defined by $\beta_{i}=\;\lambda_{i}+n-i$ where 1≤i≤n . The sequence { $\;\beta_{1},\beta_{2},\ldots ,\beta_{n}\}\;$ is called the beta-number sequence.

Example 2. ^14^Consider of Example 1, λ=(6,6,3,2,1,1) then beta number of λ is

$$\;\beta_{i}=\lambda_{i}+n-i,\;\text{where}\;i=1,2,3,4,5,6\;\text{and}\;n=6$$

And hence

$$\;\beta_{1}=11,\;\beta_{2}=10,\;\beta_{3}=6,\;\beta_{4}=4,\;\beta_{5}=2,\;\beta_{6}=1$$

Thus beta number sequence is

{11,10,6,4,2,1}.

Definition 3. ^14^Let b≥2 denote the number of columns (runners) , and let *λ* be a partition with beta number sequence $\left\{\;\beta_{1},\beta_{2},\ldots ,\beta_{n}\right\}$ . The James Abacus with b columns is a graphical representation of *λ* obtained by arranging the non negative integers 0,1, … from left to right and row by row.

A bead is placed at each position whose label belongs to the beta- number sequence of, all remaining position are left vacant.

Consider Example 1, the James Abacus with 1,2,3,4,6,7,10,11 beta position as show in next Figure 3.

Definintion 4. ^9^ The nested chain Abaus (N.C.A) treads the abacus A as the two dimensional grid of d rows and b columns. Where this N.C.A structure is utilized to represent various polyominoes configurations.

Each position on the abacus is determined by its coordinates (m,j), where 1≤m≤d, represent the row index, and 1≤j≤b represent the column index. The beta-number β corresponding to the coordinate (m,j) is given by the mapping β=(m−1)b+(j−1) .

The N.C.A structurally partitioned into finite sequence of concentric, non-over lapping, and clouded boundary path called chain denoted by $C_{1},C_{2},\ldots ,C_{r}$ where r is the number of chains such that $\overset{r}{\underset{i=1}{⋂}}C_{i}$ =∅ and $\overset{r}{\underset{i=1}{⋃}}C_{r}=A$ .

Not. For the formal construction, geometric properties $\overset{\varepsilon }{\underset{r=1}{⋂}}C_{r}$ s and coding algorithm of the N.C.A the reader referred to Reference 9.

Next Figure 4 gives an example of N.C.A. with 3 chains represented of 94, where the beta number sequence is {0,5,6,7,8,9,10,11,14,15,16,19,20,21,24}.

**Figure 3.**
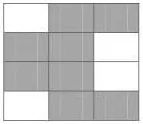
James’s Abacus with 3 columns, 4 rows and 6 beta.

**Figure 4.**
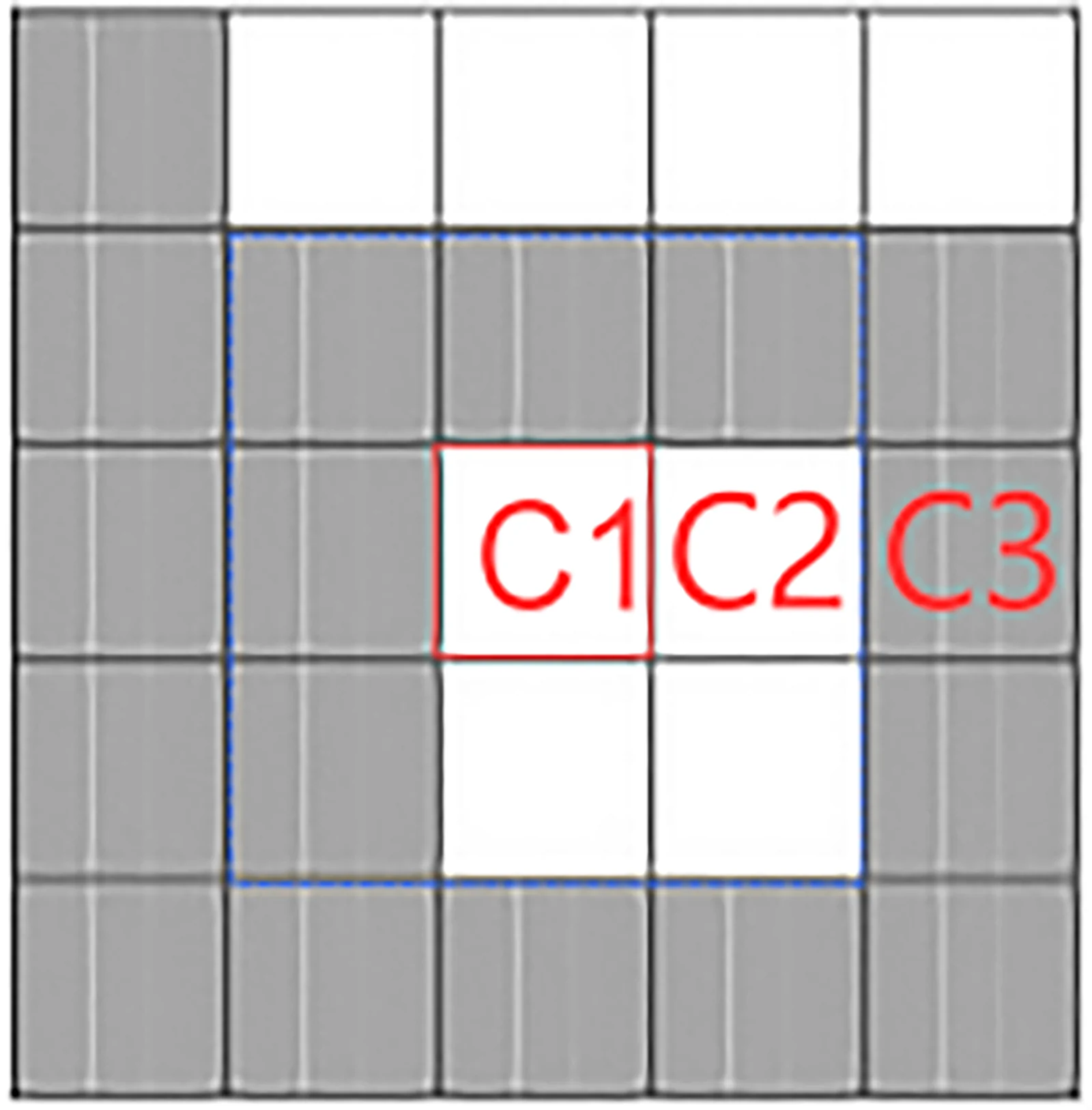
N.C.A. consisting of 5-columns and 5-rows decomposed into three nested chain ( *C*_1_, *C*_2_, *C*_3_) representing the partition of 94. The corresponding beta-number sequence is {0,5,6,7,8,9,10,11,14,15,16,19,20,21,24}. Grey cells denoted beta position whereas white cells denote the Vance positions.

Definition 5. A chain *C*_*r*_ is said to be connected chain if every position ( *m*, *j*) in *C*_*r*_ is a beta-position. Equivalenty contain no empty position.

Next Figure 5 gives an example of N.C.A. represented by a beta sequence {0,1,2,3,4,5,7,8,9,10,12,14,15,19,20,21,22,23,24} with one connected chain (chain 1).

**Figure 5.**
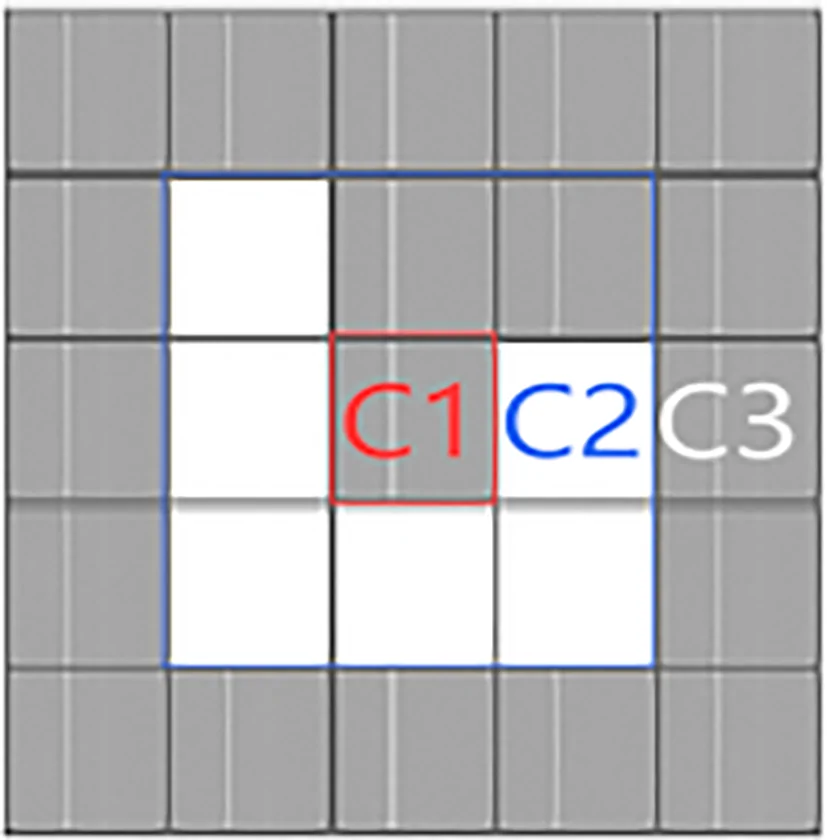
Example of N.C.A illustrating a connected third chain as defined in Definition 5.

Definition 6. Two beta numbers $\;\beta_{i}\;\text{and}\;\beta_{j}$ represented on Nested Chain Abacus with *b*-columns and *d*-rows are said to be connected iff either of the following condition is satisfied.

1-
$\left|\;\beta_{i}-\beta_{j}\;\right|=1$ provided that $\;\beta_{i},\;\beta_{j}$ are Located in the same row.
2-
$\left|\;\beta_{i}-\beta_{j}\;\right|=b\;\text{provided}\;\text{that}\;\;\beta_{i},\;\beta_{j}\;\text{are Located in the same column}$ .

For example, the beta set

{0,5,6,7,8,9,10,11,14,15,16,19,20,21,24}.

From Figure 4 is a connected.

Definition 7. Each nested chain abacus (N.C.A.) with b - columns and *d*- rows is called a polyamines if every two beta numbers are connected.

Definition 8. Onsider a Nested Chain Abacus(A) consisting of b-columns and d-rows, with chains *C*_1_, *C*_2_, … , *C*_r_ orders from innermost chain to the outermost chain where (b=d) and b is odd. The N.C.A is called Ω-nested Abacus if it satisfies the following structural condition.

1. If the unique position in C1 is occupied by a bead (beta position), then the second chain C2 contains at last five beta positions.
2. If the unique position in C1 is vacant, then the second chain C2 contains at least one beta position.
3. Every chain with an even index may contain both beta position and vacant position.
4. Every chain with an odd index greater than one forms a connected chain.

Any polyomino represented by Ω-nested Abacus is called Ω-nested Abacus polyomino.

Novelty of the Ω-nested Abacus

The proposed Ω-nested Abacus should e regarded as a restricted subclass of the N.C.A, rather than as a replacement for the original representation. While th N.C.A provide a general framework for representing arbitrary polyomines on James Abacus, it dose not impose structural constraints that facilitate systematic enumeration through local transformation.

The Ω-nested Abacus intrduses explicit condition on the parity and connectivity of the chains. In particular, every odd chain is required to remain connected, whereas every even chain is permitted to contain both occupied and empty beta positions.

Remark 9.

1- For a N.C.A with (b=d) and b is odd, the innermost chain C1 consists of a unique central position (see Reference 9).
2- The additional structural in Definition 8 distinguish the Ω -nested Abacus form the general N.C.A. These condition are imposed to ensure that the corresponding polyomino remain connected under the SSPT and MSPT transformation.

Figure 6 gives an example of Ω -nested Abacus with four chains, where

Not Every polyamines inscribed in a nested chain-abacus is Ω -nested Abacus.

Figure 4 given an example of N.C.A. but not Ω -nested Abacus, while Figure 6 given an example of Ω -nested Abacus.

Not. Throughout our result the underlying new family ( Ω -nested Abacus) assumed b=d where b is odd number. Since every odd chain is completely filled, so only even chain well be contribute to the generating process.

The algorithm construct to generate classes of new family depended on the following transformation.

Definition 9. Single – beta transformation (SPT)

Let an Ω -nested Abacus with b column, d rows and $C_{i}$ chains. Suppose that a beta number β=(m−1)b+(j−1) in chain $C_{i}$ is located in row m and column j such that 1≤m≤d , 1≤j≤b and 1≤i≤r . A single beta transformation (SPT) in chain $C_{i}$ is local transformation ( $\beta \to \overset{`}{\beta }$ ) within the same chain where

$$\overset{`}{\beta }=\{\begin{array}{ccc} \left(m-1)b+(j-2\right) & \text{if} & i+1\;\leq \;m\;\leq \;(r-i+1),\;j=b-i+1\\ \mathrm{mb}+(j-1) & \text{if} & i\;\leq \;m\;\leq \;(r-i),\;j=i\\ (m-1)b+j\; & \text{if} & m=i,\;i+1<j\;\leq \;b-i+1\\ \left(m-2)b+(j-1\right) & \text{if} & m=d-i+1,\;i\;\leq \;j<b-i. \end{array}$$

Lemma 10. The maximal number of Single Beta transformations (SPT) that can be performed in a chain *C*_i_ ( *i* > 1) is equal to the number of positions in *C*_i_.

Proof. Let C _i_ be a chain consisting of N _i_ positions. By Definition 9, a single Beta transformation acts locally on beta position and moves it to the next position within the same chain according to the prescribed transformation rule. Since every position in C _i_ has a unique successor and every position can be selected exactly once as the starting point of a SSpt, each position determines one distinct SSPT. Therefore, the total number of distinct SSPT is equal to the number of position in C _i_, namely N _i_, consequently, after N _i_ successive SSPT, every beta position returns to its original location, completing on full cycle of the chain. Hence, the maximal number of SSPT in C _i_ is equal to the number of position in that chain.

Let C _i_ (i>1) be a chain consisting of N _i_

Figure 7 illustrates Single – beta transformation (SPT)

Not.

1- SSpt is a SPT transformation in one chain.
2- MSpt is a SPT transformation in all chains.

**Figure 6.**
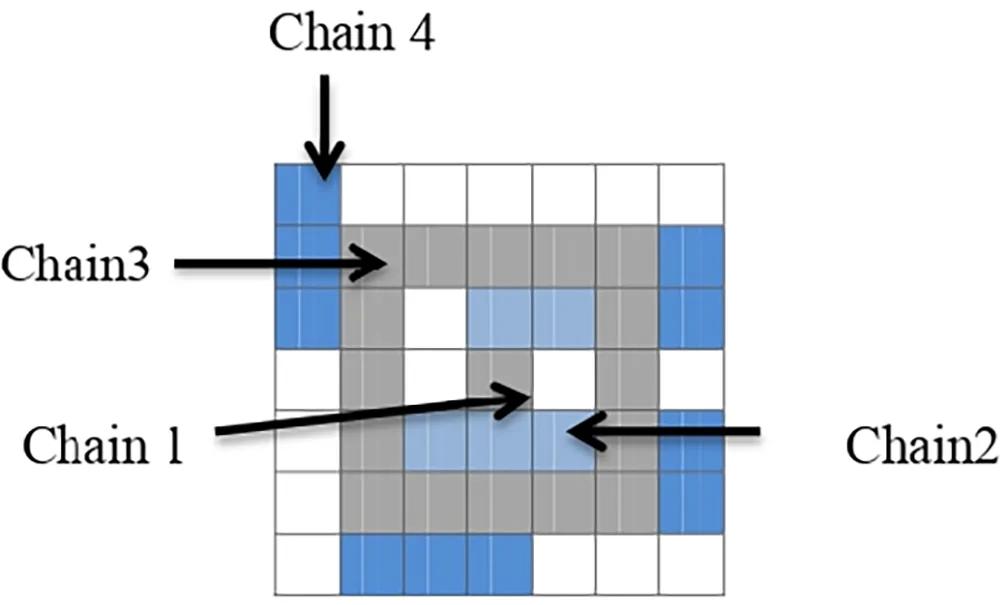
Ω -nested Abacus with four chains.

**Figure 7.**
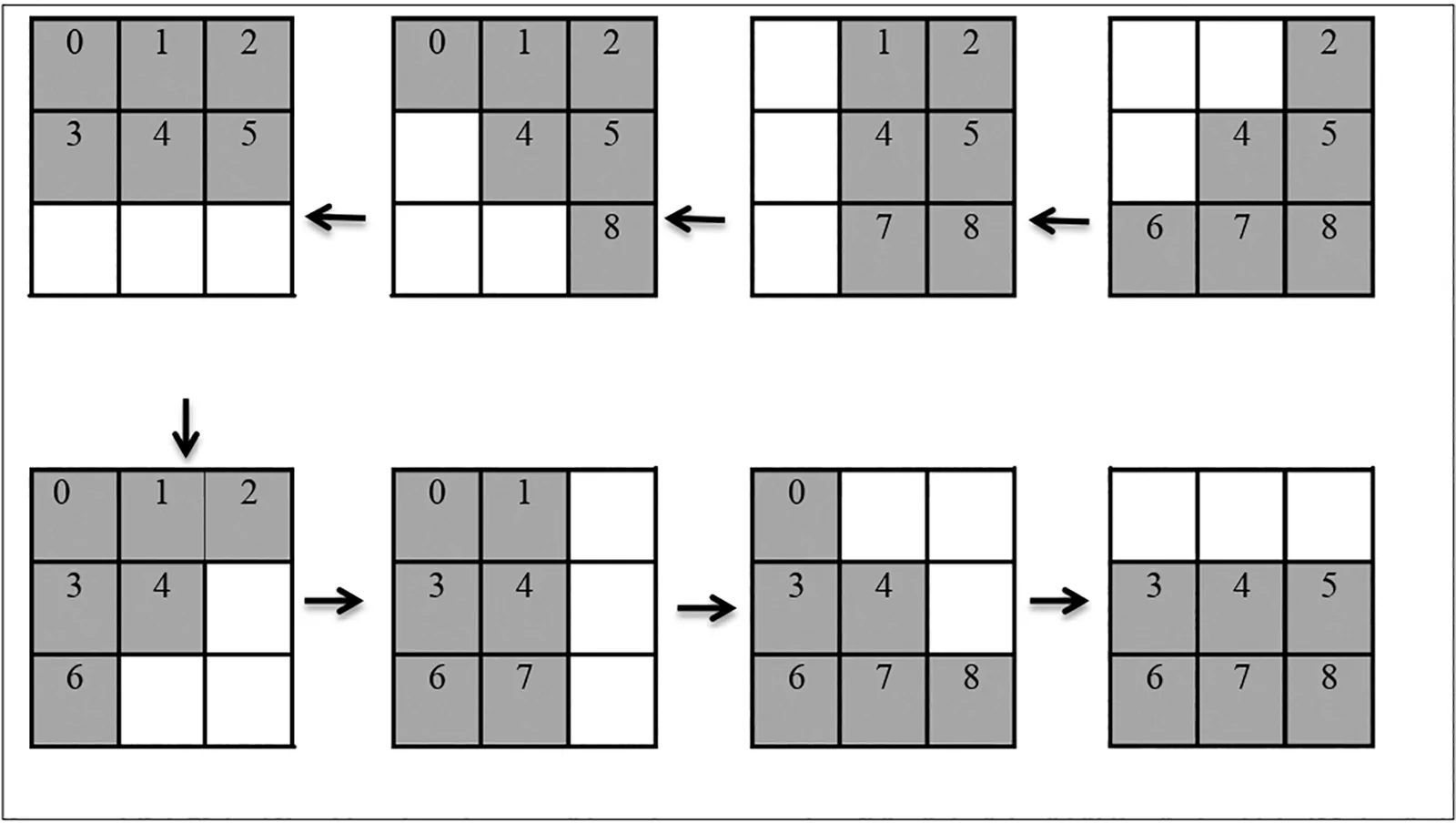
Illustrates Single – beta transformation (SPT) on Ω -nested Abacus.

## Enumeration of the Ω -nested Abacus class

Next, the enumeration of Ω-nested Abacus with *b* columns and *d* rows is presented.

Lemma 11. Let A be Ω -nested Abacus with b column, d rows and *C*_1_, *C*_2_, … , *C*_n_ then, the number of position in any chains *C*_*i*_ is $|C_{i}|=8(n-1).$

Proof. Based on Remark 8 where d=b is odd, the innermost chain $C_{1}$ colonists of a unique position. According to Definition 4, every chain $C_{n}$ (n>1) is the closed boundary of the remaining sub-grid after removing the inner chain *C*_1_, *C*_2_, … , *C*_n−1_. The second chain ( $C_{2}$ ) is formed by the eight boundary position surround the unique central position. Then

$$|C_{2}|=4+4=8$$

$$|C_{3}|=|C_{2}|+8=8.2=16$$

$$|C_{4}|=|C_{3}|+8=8.3=24\ldots \ldots \ldots |C_{n}|=|C_{n-1}|+8=8(n-1)$$

Corollary 12. Based on Remark 10 and Lemma 11, the number of (SSPT) transformation in chain $C_{n}$ is equal to 8(n−1) .

Lemma 13. Let Ω-nested Abacus be a polyomino intercept in N.C.A. with b columns and d rows such that b=d and b is odd. Then the total number of chain is $\frac{b+1}{2}\;$ .

Proof. Based on Ω-nested Abacus structure is symmetric with respect to a central column (central chain $C_{n}$ ) then for all *i*-th chains, the boundary columns must satisfy i≤b−i+1 , thus $2i\;\leq \;b-1\;\to \;i\;\leq \;\frac{b+1}{2}$ . Thus the number of even chain is $\frac{b+1}{2}$ chains.

Proposition 14. Let Ω-nested Abacus be a polyomino intercept in NCA with b columns and d rows such that b=d and b is odd. Then the number of even chain is $\frac{b-1}{4}$ .

Provided that b≡1(mod4).

Proof. Based on Lemma 13 the number of chain in Ω-nested Abacus is $t=\frac{b+1}{2}$ . The even chains among 1,2,…,t are $2,4,\ldots ,\lfloor \frac{t}{2}\rfloor$ . Since $t=\frac{b+1}{2}$ is even then $\frac{t}{2}=\frac{\frac{b+1}{2}}{2}$ is even since the first chain is odd and fixed. Then there are Since $t=\frac{b+1}{2}$ is even then $\frac{t}{2}=\frac{\frac{b+1}{2}-1}{2}=\frac{b-1}{4}$ , then the number of even chain is $\frac{b-1}{4}$ .

Next we found the number of Ω-nested Abacus if we apply SSPT-Transformation in chain i .

Lemma 15: Let Ω-nested Abacus be a polyomino intercept in NCA with b columns and d rows such that b=d , b is odd and let n be even number. Then the number of Ω-nested Abacus generating by employ SSPT-Transformation exactly chain n is 8(n−1).

Proof. By Lemma 11 the number of admissible positions in chains n is 8(n−1) . Since move each beta position yields a distinct Ω-nested Abacus under SSPT-transformation obtain in chain n with 8(n−1) position is 8(n−1) .

Theorem 16: Let Ω-nested Abacus be a polyomino intercept in N.C.A. with b columns and d rows such that b=d , b is odd and let n be even number. Then the number of Ω-nested Abacus generating by employ SSPT-Transformation is

∑∀n(R(n−1)8)

Where $R=\sum_{n}^{\left\lfloor \frac{b+1}{4}\right\rfloor }(n-1)8$ , b≡1(mod4)

Proof. Based on Lemma 10, the number of positions in chain n is (n−1)23 by Lemma 11 and Definition 8, there are b−14 chain with beta and empty beta position in Ω-nested Abacus. The maximal number of transformations in chain n is equal to ((n−1)8). Since each move generates a new Abacus (polyominoes), thus there are ((n−1)8) of Ω-nested Abacus generated by employing SSPt transformation. As a result of this, there are

∑∀n(R(n−1)8)

Ω-nested Abacus.

Theorem 17. Let Ω-nested Abacus be a polyomino intercept in N.C.A. with b columns and d rows such that b=d , b is odd and let n be even number. Then the number of Ω-nested Abacus generating by employ SSPT-Transformation exactly one even chain.

$$\sum_{n=2}^{\left\lfloor \frac{b+1}{4}\right\rfloor }8(n-1).$$

Where b≡1(mod4)

Proof. Based on Lemma 11 the number of positions in chain n is 8( *n*−1), any position in the chain will be determines one distinct SSPT in that chain. Thus all even chain $C_{2},C_{4},\ldots ,$ gives $\sum_{n=2}^{\left\lfloor \frac{b+1}{4}\right\rfloor }8(n-1)$ .

Theorem 18. Let Ω-nested Abacus be a polyomino intercept in N.C.A. with b columns and d rows such that b=d , b is odd and let n be even number. Then the number of Ω-nested Abacus generating by employ MSPT-Transformation exactly chain n is

$$\prod_{\forall n}8(n-1)$$

Where b≡1(mod4) and $n=2,4,\ldots ..,\frac{b=1}{4}$

Proof. Based on Lemma 11, the number of admissible beta and empty positions in chain C *n* is 8( *n*−1). Since all odd chains are connected and remain fixed, they do not participate in the transformation. By Lemma 15 the number of Ω-nested Abacus generating by employ SSPT-Transformation applying an SSPT to an even chain produces exactly 8( *n*−1) distinct Ω-nested Abacus. Furthermore, by Definition 4, the chains of a nested chain Abacus are pairwise disjoint. Hence, each SSPT acts locally within its own even chain and dose not affect the position of any other chain. Therefore, the transformations performed on different even chains are mutually independent and can be applied simultaneously. Consequently, an MSPT is obtained by applying an SSPT to every even chain at the same time. Since each even chain contributes independently 8(n-1) admissible transformations, the total number of Ω-nested Abacus generated after application SSPT-Transformation

$$\prod_{2\leq n\leq \frac{b-1}{2},n\;\text{even}}8\left(n-1\right)$$

## Generating function with respect to chains

In this section, the method described in ^11, 12^ is employed to enumerate the Ω-nested Abacus class, representing polyominoes inscribed within a James diagram. The ECO (Enumerating Combinatorial Objects) method has previously been applied to the enumeration of various polyomino classes. ^12^ This approach is based on a *succession rule.*

### Generating Function (G.F.)

Let $C_{2k}$ be even chain of Ω-nested Abacus, based on Lemma 11 the number of positions in any chain is

$$M_{k}=\lceil \;C_{2k}\rceil =8(n-1).$$

We apply SSPT-Transformation inside the chains such that no previously select position may be chose again. Thus, in initial stage there are $M_{k}$ choices ( $M_{k}$ of Ω-nested Abacus), after one insertion we have only $M_{k}-1$ position ( $M_{k}-1$ of Ω-nested Abacus), after two distinct insertion there are only $M_{k}-2$ position ( $M_{k}-2$ of Ω-nested Abacus as shown in Figure 8), and so on. Thus, there are

$$a_{n}^{k}=M_{k}(M_{k}-1)(M_{k}-2)\ldots (M_{k}-n+1),2\;\leq \;n\;\leq \;M_{k}$$

**Figure 8.**
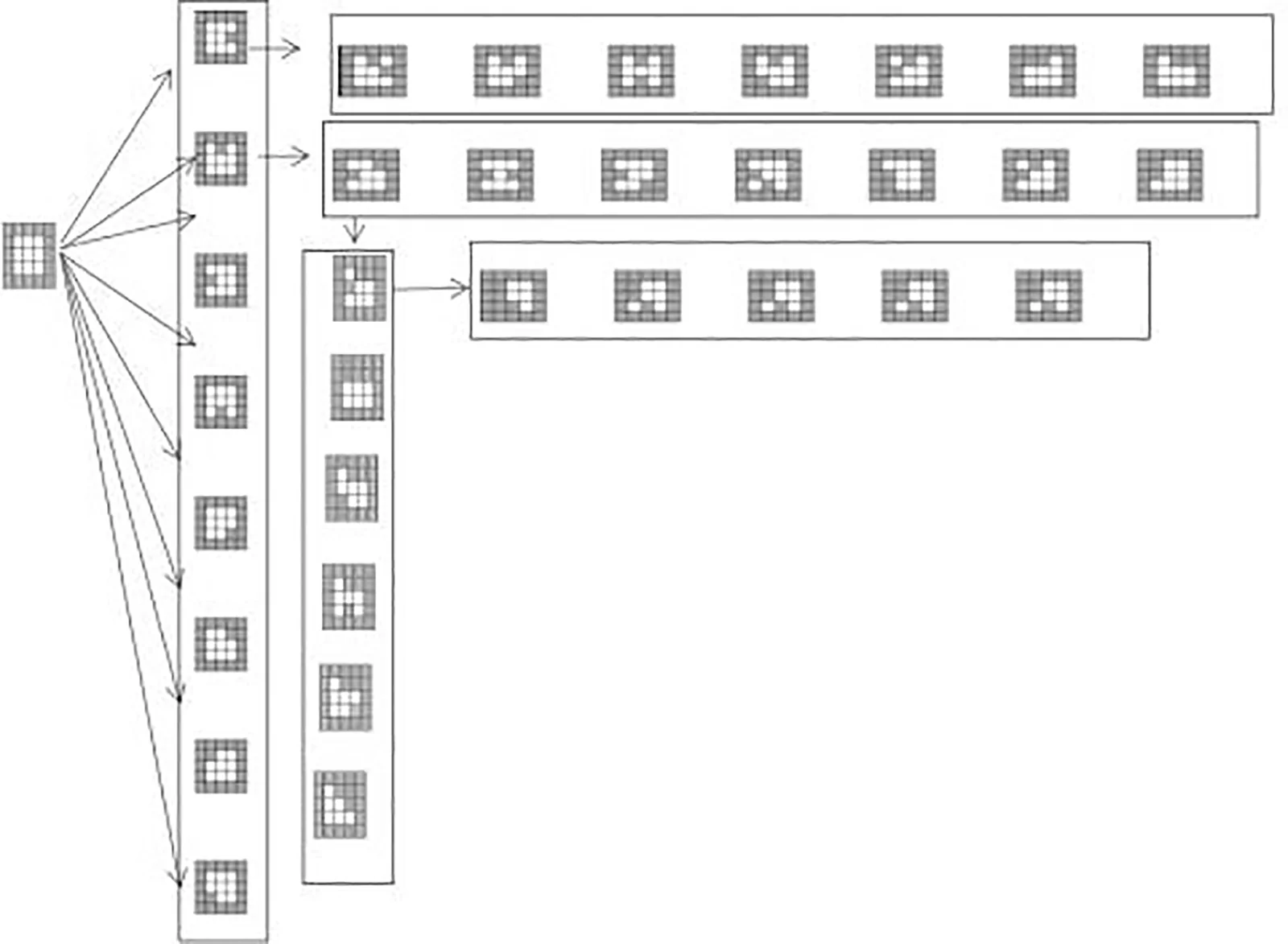
First and second level of ϑ using Ω -nested Abacus class with 5 columns, 5 rows and 3 chain.

Above process is encoded by a generating tree. The root labeled by $M_{k}$ , each node produces j children, every one of j children product j−1 . Thus the local succession rule is

$$\vartheta =\{\begin{array}{ccc} M_{k} & & \\ j & \;\to & {\left(j-1\right)}^{j}.1\;\leq \;j\;\leq \;M_{k} \end{array}$$

Assume that $a_{n+1}^{k}$ denoted the number of nodes at level n ϑ yield the recurrence

$$a_{n+1}^{k}=(M_{k}-n)a_{n}^{k}$$

Where $a_{0}^{k}=1,2\;\leq \;n\;\leq \;M_{k}$ .

$$a_{n}^{k}=\frac{M_{k}!}{(M_{k}-n)!}.$$

Using the ordinary level-generating polynomial of the even chain

$$C_{k}(x)=\sum_{n=2}^{M_{k}}(M_{k}{)}_{n}x^{n}$$

$$C_{k}(x)=\sum_{n=2}^{M_{k}}\frac{{\left(M_{k}\right)}_{n}}{n!}x^{n}$$

Since

$$\frac{{\left(M_{k}\right)}_{n}}{n!}=\frac{M_{k}!}{(M_{k}-n)!n!}=(\begin{array}{c} M_{k}\\ n \end{array})$$

then,

$$C_{k}(x)=\sum_{n=2}^{M_{k}}(\begin{array}{c} M_{k}\\ n \end{array})x^{n}=(1+x{)}^{M_{k}}$$

$$C_{k}(x)=\sum_{n=2}^{M_{k}}(\begin{array}{c} M_{k}\\ n \end{array})x^{n}=(1+x{)}^{8(2k-1)}$$

Then the generating function

$$C_{k}\left(z,x\right)=\sum_{k=1}^{m}Z^{k}{\left(1+x\right)}^{16k-8}$$

$$C_{k}\left(z,x\right)={\left(1+x\right)}^{-8}\sum_{k=1}^{m}Z^{k}{\left(1+x\right)}^{16k}$$

$$C_{k}\left(z,x\right)={\left(1+x\right)}^{-8}\sum_{k=1}^{m}{\left(Z{\left(1+x\right)}^{16}\right)}^{k}$$

$$\;\text{Let}\;\text{r}=Z{\left(1+x\right)}^{16}$$

$$C_{k}\left(z,x\right)={\left(1+x\right)}^{-8}\sum_{k=1}^{m}r^{k}$$

using the closed form sum for a finite geometric progression

$$\sum_{k=1}^{m}r^{k}=\frac{r\left(1-r^{m}\right)}{1-r}$$

Then

$$C_{k}\left(z,x\right)={\left(1+x\right)}^{-8}\;\frac{r\left(1-r^{m}\right)}{1-r}$$

$$C_{k}\left(z,x\right)={\left(1+x\right)}^{-8}\;\frac{Z{\left(1+x\right)}^{16}\left(1-{\left(Z{\left(1+x\right)}^{16}\right)}^{m}\right)}{1-Z{\left(1+x\right)}^{16}}$$

$C_{k}(z,x)$ is generating function to enumerated the number of Ω-nested Abacus.

## Conclusion

This study examines a class of polyominoes known as the Ω-nested Abacus. Initially, we provide a characterization of a new class based on specific geometric constraints defined by rows, columns, and chains. A series of operations on polyominoes is then introduced using a partition-theoretic construct known as the beta number. Furthermore, a set of operations on polyominoes is developed using a partition-theoretic construct known as the beta number, allowing for localized transformation of the structure. Furthermore, A succession rule is formulated based on generating tree techniques to describe the growth of these object. Based on this framework a recursive method is established for the systematic generation of Ω-nested Abacus configuration of a given size through the use of generating trees.

## Ethical approval

Ethical approval was not required for this study.

## Data Availability

No data availability with this atrial as the study is purely theoretical and does not involve the generating analysis or use of any datasets.
