# Supplementary material for: Enumeration Class of Polyominoes Inscribed in James Abacus and Related ECO
Source: F1000Res. 2026 Jul 30;15:11. Originally published 2026 Jan 6. [Version 3] doi: 10.12688/f1000research.172910.3 (PMC13554842; doi:10.12688/f1000research.172910.3)
Supplement: Peer review history

# Reviewer report: Reviewer response for version 2

**DOI:** 10.5256/f1000research.197917.r497664

**Published:** 2026-07-16

**Authors:** Alina Alb Lupas

**Affiliations:** 1 University of Oradea, Oradea, Romania

The paper introduces and studies a new class of polyominoes defined by unique representations on the James abacus using nested chains, termed the **Ω-nested Abacus**. It establishes structural conditions for this class and develops local transformations to analyze and enumerate the resulting combinatorial objects.

 The results are new, correct and detailed. The paper is original and doesn’t contradict to ethical or policy issues, the question posed by authors is new and well defined, the methods used by authors are appropriate and well described, the data are sound and well controlled, the discussion and conclusions are well balanced, the title and abstract convey the obtained results, the writing is acceptable, the paper contains good scientific results.

 I recommend the paper to indexing.

Is the work clearly and accurately presented and does it cite the current literature?

Yes

If applicable, is the statistical analysis and its interpretation appropriate?

Yes

Are all the source data underlying the results available to ensure full reproducibility?

Yes

Is the study design appropriate and is the work technically sound?

Yes

Are the conclusions drawn adequately supported by the results?

Yes

Are sufficient details of methods and analysis provided to allow replication by others?

Yes

Reviewer Expertise:

Mathematical analysis, complex analysis, topological algebra

I confirm that I have read this submission and believe that I have an appropriate level of expertise to confirm that it is of an acceptable scientific standard.

# Reviewer report: Reviewer response for version 2

**DOI:** 10.5256/f1000research.197917.r497661

**Published:** 2026-07-11

**Authors:** Ali A. Aubad

**Affiliations:** 1 University of Baghdad, Baghdad, Iraq

*The researchers aim to present a new class defined through a representation on the James abacus via a nested chain, which is denoted as the Ω-nested abacus. Several results are obtained within the manuscript. Despite the results obtained by the researchers, the manuscript needs to be carefully revised in its entirety, according to the comments attached below:*

**1**- *References 4 and 9 do not have the definitions* *they* *are cited to support. Use appropriate references.*

**2**- Check all the symbols that you use in the manuscript. For example, *in Definition 2, replace* *k* *with* *b* *.*

**3**- *Definitions 2, 3, and 4 are not clearly introduced. For example, Definition 3 does not specify the runner* *e* *, and the chain structures within the N.C.A. definition are unclear.*

**4**- *Figures 4 and 5 require further verification and additional detail.*

**5**- *The researchers relied on a single case study, namely the case where* *b=d* *is odd. The authors must extend their analysis to validate the generality of their findings.*

***6*** *- The authors are obliged to provide a mathematical proof for Remark 10.*

***7*** *- The proof of Lemma 11 is incomplete; it needs more details on how the final formula is obtained. The same holds for Theorem 16.*

***8*** *-* *The last sentence of the proof of Lemma 13 needs to be: “then the number of even chains is (* *b-1)/* *4* *,* *remove everything before that.*

***9*** *-* *In the proof of Theorem 18, the researchers state: 'Since the MSPT-transformation acts simultaneously on all even chains.' However, this claim requires further explanation.*

***10*** *- The researchers suggest a new family called 'Ω-nested Abacus', however, the definition of a nested abacus already exists. What improvement has been made in the researchers' definition?*

**11** *-* *There is not enough information in the 'Generating function with respect to chains' section. The proof of the G.F. formula is requested, and more details on the ECO methods are needed as well.*

***12*** *- The authors should thoroughly review the entire manuscript for language and scientific formatting.*

Is the work clearly and accurately presented and does it cite the current literature?

Partly

If applicable, is the statistical analysis and its interpretation appropriate?

Not applicable

Are all the source data underlying the results available to ensure full reproducibility?

No source data required

Is the study design appropriate and is the work technically sound?

Partly

Are the conclusions drawn adequately supported by the results?

Yes

Are sufficient details of methods and analysis provided to allow replication by others?

Partly

Reviewer Expertise:

Pure Mathematics (Algebraic Graph Theory)

I confirm that I have read this submission and believe that I have an appropriate level of expertise to confirm that it is of an acceptable scientific standard, however I have significant reservations, as outlined above.

# Response

**Published:** 2026-07-21

**Authors:** Eman Mohommed

**Affiliations:** mathematics, Mustansiriyah University Department of Mathematics, Baghdad, Baghdad Governorate, Iraq

We sincerely thank the reviewer for the careful reading of our manuscript and for the constructive comments. All remarks have been carefully considered, and the manuscript has been revised accordingly. A detailed response to each comment is provided below.

**Comment 1**

**References 4 and 9 do not contain the definitions cited by the authors.**

**Response**

 Thank you for pointing out this issue. The cited references have been carefully rechecked, and the corresponding citations have been corrected. In particular, the definitions related to the Nested Chain Abacus have been revised and now consistently refer to the appropriate source. The manuscript has also been updated to ensure that every definition is supported by the correct reference.

**Comment 2**

**The symbols should be checked. In Definition 2, the notation** k **should be replaced by** b **.**

**Response**

 The notation has been revised throughout the manuscript to ensure consistency. In particular, the parameter b is now used uniformly to denote the number of columns of the Nested Chain Abacus, while d denotes the number of rows. The notation has been checked throughout the manuscript to avoid ambiguity.

**Comment 3**

**Definitions 2, 3 and 4 require further clarification.**

**Response**

 Definitions 2, 3 and 4 have been substantially rewritten to improve clarity and mathematical precision. The structure of the Nested Chain Abacus is now explicitly defined in terms of rows, columns, chains, and beta positions. Additional explanations have been included to clarify the geometric interpretation and the role of each chain.

**Comment 4**

**Figures 4 and 5 require further verification and additional detail.**

**Response**

 The captions of Figures 4 and 5 have been revised to provide additional structural information. Figure 4 now explicitly describes the corresponding Nested Chain Abacus together with the associated beta-number sequence. Figure 5 has been presented as an independent example illustrating the notion of a connected chain, thereby supporting Definition 6 and improving the interpretation of the subsequent transformations.

**Comment 5**

**The researchers relied on a single case study, namely the case where** b=db=db=d **is odd. The authors must extend their analysis to validate the generality of their findings.**

**Response**

 The manuscript has been revised to clarify the scope of the proposed model. Rather than treating the case b=d= odd as a single example, this condition is now incorporated directly into the definition of the Ω-nested Abacus. A separate remark has also been added explaining that an odd number of rows and columns guarantees the existence of a unique central position, which is an essential structural requirement for the proposed class. Consequently, all subsequent lemmas and theorems are formulated for the entire family of Ω-nested Abaci satisfying this condition, rather than for a single illustrative example.

**Comment 6**

**The authors are obliged to provide a mathematical proof for Remark 10.**

**Response**

 A complete mathematical justification has been added. The revised proof explicitly shows that each admissible position in a chain produces exactly one SSPT-transformation and that every position is transformed at most once. Consequently, the maximal number of SSPT-transformations is equal to the number of positions in the chain.

**Comment 7**

**The proof of Lemma 11 is incomplete; it needs more details on how the final formula is obtained. The same holds for Theorem 16.**

**Response**

 The proof of Lemma 11 has been expanded by explicitly explaining the geometric growth of successive chains. The derivation now shows why the second chain contains exactly eight positions and how each additional chain increases the boundary by eight positions, leading to the formula

 ∣C _n_∣=8(n−1)

 Similarly, the proof of Theorem 16 has been rewritten to provide the missing intermediate arguments and improve the logical flow.

**Comment 8**

**The last sentence of the proof of Lemma 13 needs to be revised.**

**Response**

 The proof has been simplified according to the reviewer's suggestion. The unnecessary sentence has been removed, and the proof now concludes directly with the statement that the number of even chains is

 ​,

 which follows immediately from the chain indexing.

**Comment 9**

**The statement "Since the MSPT-transformation acts simultaneously on all even chains" requires further explanation.**

**Response**

 Additional justification has been incorporated into the proof of Theorem 18. The revised proof now explains that the chains of the Nested Chain Abacus are pairwise disjoint; therefore, each SSPT acts locally within its own even chain and does not affect any other chain. Consequently, the admissible transformations in different even chains are independent, allowing the MSPT-transformation to be interpreted as the simultaneous application of SSPTs to all even chains. The final counting formula follows directly from the multiplication principle.

**Comment 10**

**There is not enough information in the "Generating function with respect to chains" section. The proof of the generating function and more details on the ECO method are required.**

**Response**

 This section has been substantially expanded. Additional material describing the ECO methodology, the generating tree, and the succession rule has been included. The recurrence relation has been derived explicitly, followed by a detailed derivation of the generating polynomial using the binomial theorem. Furthermore, the derivation of the bivariate generating function has been expanded by including the omitted intermediate algebraic steps and the application of the finite geometric-series identity. These revisions provide a complete mathematical justification of the generating function while preserving the original enumeration results.

**Final Remark**

 We are grateful to the reviewer for the valuable comments and suggestions. We believe that the revisions have significantly improved the mathematical rigor, clarity, and presentation of the manuscript, and we hope that the revised version satisfactorily addresses all concerns raised during the review process.

# Reviewer report: Reviewer response for version 2

**DOI:** 10.5256/f1000research.197917.r485075

**Published:** 2026-05-25

**Authors:** Hayder R. Hashim

**Affiliations:** 1 University of Kufa, Kufa, Iraq

The authors have revised the manuscript in accordance with my comments. My only remaining suggestion to the authors is to carefully polish the language. Otherwise, I recommend acceptance for indexing.

Is the work clearly and accurately presented and does it cite the current literature?

Yes

If applicable, is the statistical analysis and its interpretation appropriate?

Not applicable

Are all the source data underlying the results available to ensure full reproducibility?

No source data required

Is the study design appropriate and is the work technically sound?

Partly

Are the conclusions drawn adequately supported by the results?

Yes

Are sufficient details of methods and analysis provided to allow replication by others?

No

Reviewer Expertise:

Number Theory and cryptography

I confirm that I have read this submission and believe that I have an appropriate level of expertise to confirm that it is of an acceptable scientific standard.

# Reviewer report: Reviewer response for version 1

**DOI:** 10.5256/f1000research.190675.r462385

**Published:** 2026-03-05

**Authors:** Hayder R. Hashim

**Affiliations:** 1 University of Kufa, Kufa, Iraq

The manuscript proposes a new class of polyominoes defined by a graphical representation referred to as the James Abacus or Nested Chain Abacus and attempts to enumerate this class using combinatorial transformations and the ECO  method.

 Since the results in the paper are interesting in number theory, I suggest accepting the paper after revising it according to the following remarks as the paper would benefit from improvements in clarity, organization, and presentation.

 1.  Lemma 11 and Theorem 12 refer to external theorems without clearly stating assumptions or applicability.

 2. Corollary 13 and Theorem 14 contain inconsistent notation  such as mixing  b,p,r,e,d  and unclear counting logic.

 3. Polish the language carefully. 

 4. Every lemma and theorem must include a complete, self-contained proof.

 5.  All variables must be clearly defined and used consistently.

 6. If results rely on previous publications, the exact statements must be cited and restated.

 7. Try to reconstruct in more details the ECO method application properly.

 8. Redo or rewrite  the derivation of the generating function theorem  from first principles, with a more clear form.

Is the work clearly and accurately presented and does it cite the current literature?

Yes

If applicable, is the statistical analysis and its interpretation appropriate?

Not applicable

Are all the source data underlying the results available to ensure full reproducibility?

No source data required

Is the study design appropriate and is the work technically sound?

Partly

Are the conclusions drawn adequately supported by the results?

Yes

Are sufficient details of methods and analysis provided to allow replication by others?

No

Reviewer Expertise:

Number Theory

I confirm that I have read this submission and believe that I have an appropriate level of expertise to confirm that it is of an acceptable scientific standard, however I have significant reservations, as outlined above.

# Response

**Published:** 2026-03-19

**Authors:** Eman Mohommed

**Affiliations:** mathematics, Mustansiriyah University Department of Mathematics, Baghdad, Baghdad Governorate, Iraq

We  thank the reviewer for his comments

 Comment 1:

 Lemma 11 and theorem 12 refer to external theorems without clearly stating assumptions of applicability

 Response: in the revised version, we have explicitly stated all required assumptions and clarified the applicability of the referenced result. In addition, we have added several lemmas and theorems related with lemma 11 and theorem 12 to ensure that lemma 11 and theorem 12 are fully self-contained and mathematically precise.

 Comment 2:

 Corollary 13 and Theorem 14 contain inconsistent notation, such as mixing b, p, r, e, d, and unclear counting logic

 Response: we appreciate this observation. The notation has been carefully revised and unified throughout the paper. All variables are now clearly defined and used consistently.

 Comment 3:

 Polish the language carefully

 Response: all manuscripts have been proofread and language has been improved.

 Comment 4:

 Every lemma and theorem must include a complete, self-contained proof.

 Response: In the new version, all lemmas and theorems now include complete proof. Additional explanations have been added where necessary.

 Comment 5:

 All variables must be clearly defined and used consistently.

 Response:

 All variables have been systematically reviewed and defined at their first occurrence and then has been standardized across the paper to ensure consistency.

 Comment 6:

 If results rely on previous publications, the exact statements must be cited and restated

 Response: we revised all relevant sections to explicitly cite the original source.

 Comment 7,8:

 Try to reconstruct in more detail the proper application of the ECO method.

 Redo or rewrite the derivation of the generating function theorem from first.

 Response: following the reviewer's suggestion, we have completely rewritten the derivation of the generating function.

# Reviewer report: Reviewer response for version 1

**DOI:** 10.5256/f1000research.190675.r450050

**Published:** 2026-01-16

**Authors:** Ayesha Qureshi

**Affiliations:** 1 Sabanci Universitesi (Ringgold ID: 52991), Istanbul, Istanbul, Turkey

This paper introduces a class of polyominoes defined via a nested chain abacus representation and aims to enumerate this class using transformations and the ECO method. While the topic may be of interest, the manuscript in its current form lacks sufficient mathematical clarity and rigor.

 Several definitions are unclear or inconsistent, and many results are stated without complete or verifiable proofs. In particular, the use of the ECO method and the derivation of the generating function require a more precise and rigorous formulation. Additionally, the presentation and language need substantial improvement. A major revision addressing these issues would be necessary before the conclusions can be considered supported by the results.

Is the work clearly and accurately presented and does it cite the current literature?

Partly

If applicable, is the statistical analysis and its interpretation appropriate?

Not applicable

Are all the source data underlying the results available to ensure full reproducibility?

No source data required

Is the study design appropriate and is the work technically sound?

Partly

Are the conclusions drawn adequately supported by the results?

No

Are sufficient details of methods and analysis provided to allow replication by others?

No

Reviewer Expertise:

Algebra

I confirm that I have read this submission and believe that I have an appropriate level of expertise to state that I do not consider it to be of an acceptable scientific standard, for reasons outlined above.
